# Trimethylbenzol (alle Isomere): MAK-Begründung, Nachtrag

**DOI:** 10.34865/mb2555113ismd11_2ad

**Published:** 2026-06-30

**Authors:** Andrea Hartwig, MAK Commission

**Affiliations:** 1 Institut für Angewandte Biowissenschaften, Abteilung Lebensmittelchemie und Toxikologie, Karlsruher Institut für Technologie (KIT) Adenauerring 20a, Geb. 50.41 76131 Karlsruhe Deutschland; 2 Ständige Senatskommission zur Prüfung gesundheitsschädlicher Arbeitsstoffe, Deutsche Forschungsgemeinschaft, Kennedyallee 40, 53175 Bonn, Deutschland. Weitere Informationen: Ständige Senatskommission zur Prüfung gesundheitsschädlicher Arbeitsstoffe | DFG

**Keywords:** Trimethylbenzol (alle Isomere), MAK-Wert, maximale Arbeitsplatzkonzentration, Neurotoxizität, Toxizität, Lunge, Reizwirkung, Spitzenbegrenzung

## Abstract

The German Senate Commission for the Investigation of Health Hazards of Chemical Compounds in the Work Area (MAK Commission) summarized and re-evaluated the data for trimethylbenzene, all isomers [95-63-8; 108-67-8; 526-73-8; 25551-13-7] to derive an occupational exposure limit value (maximum concentration at the workplace, MAK value) considering all toxicological end points. Relevant studies were identified from a literature search. The critical effect is preclinical neurotoxicity. A NOAEC of 25 ml/m^3^ was established in rats after inhalation exposure to 1,2,4-trimethylbenzene and 1,2,3-trimethylbenzene based on reduced performance in the rotarod test for motor coordination and prolonged latency times in the hot plate test. A MAK value of 5 ml/m^3^ was derived for trimethylbenzene on this basis. The assignment to Peak Limitation Category II with an excursion factor of 2 has been retained. The tumour incidence was not increased in male and female Sprague Dawley rats after 2-year gavage administration (4 days/week) of 1,2,4-trimethylbenzene doses of 800 mg/kg body weight per day. Trimethylbenzene was not mutagenic or clastogenic in vitro and did not induce micronuclei in vivo. Data for germ cells are not available. Therefore, trimethylbenzene (all isomers) has not been classified in a category for germ cell mutagens. No teratogenic potential can be inferred from the available studies that investigated developmental toxicity in rats and mice and trimethylbenzene has been assigned to Pregnancy Group D. Additionally, there are no experimental data showing that juvenile animals are more sensitive to trimethylbenzene-induced neurotoxic effects than adults. Therefore, there is no evidence that would require re-categorization of the substance from Pregnancy Group D to Pregnancy Group B (suspected). Skin contact is not expected to contribute significantly to systemic toxicity and there are no indications of sensitizing effects.

**Table:** 

**MAK-Wert (2025)**	**5 ml/m^3^ (ppm) ≙ 25 mg/m^3^**
**Spitzenbegrenzung (2001)**	**Kategorie II, Überschreitungsfaktor 2**
	
**Hautresorption**	**–**
**Sensibilisierende Wirkung**	**–**
**Krebserzeugende Wirkung**	**–**
**Fruchtschädigende Wirkung (2025)**	**Gruppe D **
**Keimzellmutagene Wirkung **	**–**
	
**BAT-Wert (2008)**	**400 mg Dimethylbenzoesäuren** **(Summe aller Isomeren nach Hydrolyse)/g Kreatinin**
	
CAS-Nr.	1,2,4-Trimethylbenzol: 95-63-8 1,3,5-Trimethylbenzol: 108-67-8 1,2,3-Trimethylbenzol: 526-73-8 Trimethylbenzol (alle Isomere): 25551-13-7
Molmasse	120,19 g/mol
Löslichkeit	75 mg 1,2,4-Trimethylbenzol/l Wasser (Myhre und Fonnum 2001) 57 mg 1,2,4-Trimethylbenzol/l Wasser 48,2 mg 1,3,5-Trimethylbenzol/l Wasser 75,2 mg 1,2,3-Trimethylbenzol/l Wasser (US EPA 2016)
	
**1 ml/m^3^ (ppm) ≙ 5 mg/m^3^**	**1 mg/m^3^ ≙ 0,2 ml/m^3^ (ppm)**
	
Hydrolysestabilität	k. A.
Verwendung	Lösungsmittel, 1,2,4-Trimethylbenzol wird zur Synthese eingesetzt (ECHA 2023)

Seit dem Jahr 2016 berücksichtigt die Kommission bei Stoffen, deren MAK-Wert auf systemischen Effekten basiert und aus inhalativen Tierversuchen oder Probandenstudien in Ruhe abgeleitet wurde, dass das Atemvolumen am Arbeitsplatz höher ist als unter diesen experimentellen Bedingungen. Dies gilt jedoch nicht für Gase und Dämpfe, wenn deren Blut:Luft-Verteilungskoeffizient < 5 ist (DFG 2025, Abschnitt I b und I c). Die mittleren Blut:Luft-Verteilungskoeffizienten der Trimethylbenzol-Isomere betragen nach Messungen 43 (1,3,5-Trimethylbenzol), 59,1 (1,2,4-Trimethylbenzol) und 66,5 (1,2,3-Trimethylbenzol) (Järnberg und Johanson 1995). Mit diesem Nachtrag wird überprüft, ob aufgrund des höheren Atemvolumens am Arbeitsplatz der MAK-Wert und die Schwangerschaftsgruppe von Trimethylbenzol geändert werden müssen.

Es liegen bisher eine Begründung (Henschler 1986) und zwei Nachträge (Greim 1998, 2001) vor.

Die drei Isomere von Trimethylbenzol sowie die aromatischen C9-Kohlenwasserstoff-Lösungsmittel zeigen ähnliche toxische Effekte, die auch in gleich hohen Konzentrations- bzw. Dosisbereichen auftreten. Damit wird von einer ähnlichen Wirksamkeit ausgegangen und die drei Trimethylbenzol-Isomere werden gemeinsam bewertet.

Die drei Trimethylbenzol-Isomere kommen in Erdöl vor und sind in Erdölraffinerien häufige Bestandteile von Destillationsfraktionen wie Testbenzin, Naphtha mit hohem Flammpunkt und Benzin (OEHHA 2023).

## 1 Allgemeiner Wirkungscharakter

Der kritische Effekt von Trimethylbenzol ist die Neurotoxizität. Bei Ratten bewirkt 1,2,3-Trimethylbenzol nach 13-wöchiger Exposition ab 100 ml/m^3^ eine eingeschränkte Koordination im Rotarod-Test und 1,2,4-Trimethylbenzol eine geringere Schmerzempfindlichkeit im Hot-Plate-Test.

Nach dermaler Applikation von unverdünntem 1,2,4- oder 1,3,5-Trimethylbenzol treten irritative Effekte bei Kaninchen auf. Trimethylbenzol zeigt eine leicht reizende Wirkung am Kaninchenauge. Es liegen keine belastbaren Hinweise auf eine sensibilisierende Wirkung vor.

Entwicklungstoxische Effekte, einschließlich Teratogenität, treten bei den Studien erst bei maternaltoxischen Konzentrationen ab 300 ml/m^3^ auf. In einer Mehrgenerationenstudie führt eine Konzentration von 103 ml C9-Aromaten-Gemisch/m^3^ (57 ml Gesamt-Trimethylbenzol/m^3^) an juvenilen Ratten zu einer deutlich verzögerten Körpergewichtsentwicklung.

Bei In-vitro- und In-vivo-Studien sind keine genotoxischen Effekte aufgetreten, außer einer gering erhöhten (1,5-fach) Anzahl an Schwesterchromatidaustauschen (SCE)/Zelle in Knochenmarkszellen von männlichen BALB/c-Mäusen nach intraperitonealer Applikation von 730 mg 1,2,3-Trimethylbenzol/kg KG, 900 mg 1,2,4-Trimethylbenzol/kg KG oder 1800 mg 1,3,5-Trimethylbenzol/kg KG.

In einer Langzeitstudie mit oraler Gabe von 800 mg 1,2,4-Trimethylbenzol/kg KG und Tag an Ratten hat sich die Tumorhäufigkeit nicht statistisch signifikant erhöht.

## 2 Wirkungsmechanismus

Die Trimethylbenzol-Isomere induzieren Cytochrom-P450-Enzyme (CYP) und damit ihren eigenen Metabolismus (Greim 1998). Durch dreitägige In-vivo-Behandlung mit 5 mM **1,3,5-Trimethylbenzol**/kg KG und Tag wurde eine Aktivitätserhöhung verschiedener Enzyme in Rattenlebermikrosomen beobachtet, dies betraf u. a. die Anilin hydroxylierenden (CYP2E1) und Ethanol oxidierenden Enzyme sowie NADPH-Cytochrom-c-Reduktase (Greim 1998; Zajworoniuk und Rzeczycki 1992).

Die Erhöhung des relativen Lebergewichts bei hohen Trimethylbenzol-Expositionen passt zu den bereits gut etablierten Merkmalen einer adaptiven Leberreaktion auf Chemikalien-Exposition (Adenuga et al. 2014).

Humane bronchiale BEAS-2B-Zellen wurden in einem „air liquid interface“ (ALI) System jeweils eine Stunde an einem, drei oder fünf Tagen gegen 0, 20 oder 100 ml **1,3,5-Trimethylbenzol**/m^3^ (Reinheit 99,9 %) exponiert. Eine mehr als zweifache Genexpression wurde bei *CYP2E1*, das auf der Proteinebene reguliert wird, mit einer zeitabhängigen Verstärkung beobachtet. Nur bei Exposition gegen 100 ml/m^3 ^war die Expression von *CYP1A1* minimal erhöht. Eine Genexpressionsinduktion von *CYP1B1* und *CYP2F1* und des Transmembran-Proteins *MUC1* trat nicht auf. Weitere statistisch signifikante Anstiege mit deutlicher zeitabhängiger Verstärkung zeigte die Expression der Aldehyddehydrogenasen *ALDH2* und *ALDH3B1* (Méausoone et al. 2021). Die Induktion des humanen *CYP2A13* wurde nicht untersucht.

Humane neutrophile Granulozyten wurden mit 640 µmol **1,2,4-Trimethylbenzol** inkubiert. Eine zeitabhängige Zunahme an reaktiven Sauerstoffspezies (ROS) wurde in den ersten 30 Minuten nach der Zugabe durch Fluoreszenzmessungen mit 2′,7′-Dichlorfluorescein (DCF) nachgewiesen. Eine geringe Zunahme von Superoxid-Anionen, die nach 200 Minuten ein Maximum erreichte, wurde mit Elektronenspinresonanz (ESR)-Spektroskopie bestimmt. Die Hydroxylierung von 4-Hydroxybenzoesäure diente als Nachweis der Entstehung von OH-Radikalen; hier konnte ebenfalls eine statistisch signifikante Steigerung nach zweistündiger Inkubation mit 640 µmol 1,2,4-Trimethylbenzol beobachtet werden. Damit zeigte 1,2,4-Trimethylbenzol eine stimulierende Wirkung auf die ROS-Produktion in neutrophilen Granulozyten (Myhre et al. 2000).

Primäre Gehirnsynaptosomen aus der Großhirnrinde von männlichen Wistar-Ratten wurden mit 0, 320, 640 oder 1280 µmol **1,2,4-Trimethylbenzol** inkubiert. Die beobachtete Zunahme der Fluoreszenz nach Zugabe von DCF war statistisch signifikant und weist nach Meinung der Autoren auf einen konzentrationsabhängigen Anstieg an ROS und reaktiven Stickstoffspezies (RNS) hin. Fluoreszenz wurde kontinuierlich 90 Minuten lang nach Beginn der Inkubation gemessen (Myhre und Fonnum 2001).

Für eine Epoxidbildung stehen beim 1,2,3-Trimethylbenzol zwei Reaktionsstellen zur Verfügung, beim 1,2,4-Trimethylbenzol eine Stelle und 1,3,5-Trimethylbenzol kann nicht epoxidiert werden.

**1,3,5-** und **1,2,4-Trimethylbenzol** waren jeweils nur in einem von 819 In-vitro-Tests der ToxCast-Assay-Suite positiv; **1,2,3-Trimethylbenzol** in einem von 216 Tests (US EPA 2025 a, b, c). Alle drei Substanzen zeigen jedoch hohe Dampfdrücke. Mittels NMR-spektroskopischer Analyse konnte in frisch in Dimethylsulfoxid gelösten Proben von **1,3,5-** und **1,2,4-Trimethylbenzol** keine bzw. nur 20 % der erwarteten Prüfsubstanzmenge detektiert werden (NIH 2025 a, b). Somit ist nicht klar, ob die inaktiven Tests auf fehlende Aktivität der Prüfsubstanzen oder fehlende Substanz in der Probe zurückzuführen sind und die Daten sind somit mit Vorsicht zu betrachten.

Zum Isomerengemisch liegen keine Daten vor.

Alkylbenzole wie Toluol und Xylol bewirkten eine Veränderung der Konzentration und des Umsatzes von Dopamin und Noradrenalin im Gehirn von Ratten und zeigten damit einen Einfluss auf das katecholaminerge System. Weitere Neurotransmitter-vermittelte Reaktionen und -Rezeptoren wurden von Alkylbenzolen sowohl hemmend als auch anregend beeinflusst. Diese neurotoxischen Wirkungen können auch für Trimethylbenzol vermutet werden. Bei sehr hohen Expositionen ist auch eine Wechselwirkung mit der Lipidmembran anzunehmen (US EPA 2016).

## 3 Toxikokinetik und Metabolismus

### 3.1 Aufnahme, Verteilung, Ausscheidung

Die Öl:Luft-Verteilungskoeffizienten betragen 9,88 (1,3,5-Trimethylbenzol), 10,2 (1,2,4-Trimethylbenzol) und 10,9 (1,2,3-Trimethylbenzol). Aufgrund dieser Ergebnisse wird eine Anreicherung von Trimethylbenzol-Isomeren im Fettgewebe erwartet (Järnberg und Johanson 1995).

Einen Vergleich der gemessenen Blut:Luft-Verteilungskoeffizienten der Trimethylbenzol-Isomere bei Mensch und Ratte zeigt Tabelle 1 (Meulenberg und Vijverberg 2000).

**Tab. 1:** Blut:Luft-Verteilungskoeffizienten (Meulenberg und Vijverberg 2000)

**Isomer**	**Mensch**	**Ratte**
1,3,5-Trimethylbenzol	43,0	55,7
1,2,4-Trimethylbenzol	59,1	57,7
1,2,3-Trimethylbenzol	66,5	62,6

#### 3.1.1 Inhalative Aufnahme

##### 3.1.1.1 Mensch

In der Lunge von fünf (vermutlich nur männlichen) Probanden wurden 68 % der eingeatmeten Menge von **1,2,4-Trimethylbenzol**, 67 % von **1,3,5-Trimethylbenzol** und 71 % von **1,2,3-Trimethylbenzol** bei vierstündiger Exposition gegen 20 oder 30 ml/m^3^ retiniert. Das Atemminutenvolumen und die exhalierte Trimethylbenzol-Konzentration wurden gemessen (Kostrzewski et al. 1997).

Bei vierstündiger Monoexposition gegen 25 ml **1,3,5-Trimethylbenzol/**m^3^ betrug die Retention ca. 23–56 % bei jeweils zwei weiblichen und männlichen Probanden. Hierbei wurde das Atemminutenvolumen allometrisch berechnet und mit der ausgeschiedenen Metabolitenmenge im Urin verglichen (Jones et al. 2006). Diese Studie ist daher nicht so verlässlich wie die von Kostrzewski et al. (1997).

Die Halbwertszeiten der Trimethylbenzole im Blut von Probanden betrugen 0,02–0,04 Stunden (Phase I), 0,33–1,7 Stunden (Phase II) und 28,9–46,2 Stunden (Phase III) (Kostrzewski et al. 1997).

An verschiedenen Tagen wurden zehn männliche Probanden zwei Stunden lang in einer Expositionskammer nacheinander gegen 25 ml **1,2,4-Trimethylbenzol**/m^3^, 25 ml **1,2,3-Trimethylbenzol**/m^3^, 25 ml **1,3,5-Trimethylbenzol**/m^3^ oder 2 ml 1,2,4-Trimethylbenzol/m^3^ exponiert und verrichteten dabei konstant 50 W Arbeit auf einem Fahrradergometer, wobei pro Tag nur eine Substanz eingesetzt wurde. Bei den drei Isomeren traten im zeitlichen Verlauf ihrer Konzentrationen im Blut keine wesentlichen Unterschiede auf. Die respiratorische Aufnahme betrug für 1,2,3-Trimethylbenzol 56 %, für 1,3,5-Trimethylbenzol 62 % und für 1,2,4-Trimethylbenzol 64 %. 20–37 % der aufgenommenen Menge wurde unverändert abgeatmet. Die Blutclearance betrug 0,63–0,97 l/Stunde/kg KG. Innerhalb von 24 Stunden wurden 22 % der inhalierten Menge von 1,2,4-Trimethylbenzol hauptsächlich als 3,4-Dimethylhippursäure mit dem Urin ausgeschieden. Von 1,2,3-Trimethylbenzol wurden innerhalb der ersten 24 Stunden nur 11 % als 2,3-Dimethylhippursäure gefunden, bei 1,3,5-Trimethylbenzol nur 3 % als 3,5-Dimethylhippursäure. Die Halbwertszeit der Ausscheidung der Dimethylhippursäure-Isomere lag zwischen vier und 16 Stunden (Greim 1998; Järnberg et al. 1996, 1997 b).

Zur Untersuchung, ob Trimethylbenzole oder ihre Metaboliten als geeignete Biomarker für eine Exposition gegen Testbenzin („white spirit“) verwendet werden können, wurden neun männliche Probanden zwei Stunden lang in einer Expositionskammer gegen 300 mg **Testbenzin**/m^3^ bei Belastung mit 50 W am Fahrradergometer exponiert. Der Anteil an 1,2,4-Trimethylbenzol dieses Testbenzins betrug 3,8 % (11 mg/m^3^, ca. 2 ml/m^3^). Die Ergebnisse wurden mit dem Ergebnis nach alleiniger Exposition gegen 11 mg 1,2,4-Trimethylbenzol/m^3^ (siehe Järnberg et al. 1996, 1997 b) verglichen. Ausatemluft, Kapillarblut und Urin wurden vor, während und nach der Exposition gesammelt und analysiert. In den ersten drei Stunden waren die Blutspiegel und die „area under the curve“ (AUC) von 1,2,4-Trimethylbenzol statistisch signifikant erhöht (ca. 65 %). Auch die kumulative Ausscheidung von 3,4-Dimethylhippursäure (Messzeitraum 0–6 Stunden nach Exposition) lag nach Exposition gegen Testbenzin statistisch signifikant höher (ca. 65 %) als bei Exposition gegen reines 1,2,4-Trimethylbenzol/m^3^. Die Autoren gehen davon aus, dass die Metabolisierung von 1,2,4-Trimethylbenzol durch weitere Komponenten des Testbenzins gehemmt wurde (Greim 1998; Järnberg et al. 1997 a).

Neun männliche Probanden, die bereits ein Jahr früher gegen **1,2,4-Trimethylbenzol** exponiert waren (siehe Järnberg et al. 1996, 1997 b), wurden zwei Stunden lang in einer Expositionskammer gegen 11 mg 1,2,4-Trimethylbenzol/m^3^ (ca. 2 ml/m^3^) in 300 mg Testbenzin („white spirit“)/m^3^ bei Belastung mit 50 W am Fahrradergometer exponiert. Die AUC von Trimethylbenzol im Blut lag bei 157 µM × Minute. Die Halbwertszeit im Blut betrug 5,9 Minuten in der ersten Phase und 4,8 Stunden in der 2. Phase. Innerhalb von sechs Stunden wurden 18 % der resorbierten Menge von 1,2,4-Trimethylbenzol als 3,4-Dimethylhippursäure mit dem Urin ausgeschieden mit einer Halbwertszeit von 3,0 Stunden. Die Autoren geben an, dass alle Daten aus den früheren Studien reanalysiert wurden. Ein Vergleich mit der oben beschriebenen Studie mit Exposition gegen reines Trimethylbenzol zeigte, dass der Trimethylbenzol-Gehalt und die AUC im Blut statistisch signifikant höher waren nach Exposition gegen 2 ml/m^3^**1,2,4-Trimethylbenzol in Testbenzin** als nach Exposition gegen 2 ml reines **1,2,4-Trimethylbenzol**/m^3^. Die metabolische Blut-Clearance war entsprechend höher bei Exposition gegen 2 ml/m^3^ allein. Dies könnte auf eine Hemmung der metabolischen Elimination durch im Testbenzin vorhandene Substanzen zurückgeführt werden. Die relative kumulative Ausscheidung des Metaboliten 3,4-Dimethylbenzoesäure (DMBA) lag statistisch signifikant höher wenn gegen 2 ml/m^3^ 1,2,4-Trimethylbenzol in Testbenzin exponiert wurde, im Vergleich zur Exposition gegen 2 oder 25 ml reines 1,2,4-Trimethylbenzol/m^3^ (Reinheit > 99 %) (Järnberg et al. 1998). Die Aussage der Autoren, dass die verringerte Metabolisierung für die geringeren Blutspiegel von 1,2,4-Trimethylbenzol im Blut verantwortlich ist, passt nicht zu der höheren ausgeschiedenen Menge des Metaboliten 3,4-DMBA.

In einer Expositionskammerstudie wurden vier Probanden vier Stunden lang gegen 25 ml **1,3,5-Trimethylbenzol**/m^3^ exponiert. 1,3,5-Trimethylbenzol wurde schnell in den Blutkreislauf aufgenommen und erreichte eine mittlere Konzentration von 0,85 µmol/l während der vierstündigen Exposition. Die Ausscheidung in der Atemluft verlief biphasisch mit einer initialen Halbwertszeit von 60 Minuten. Es zeigte sich auch eine biphasische Ausscheidung von 3,5-Dimethylhippursäure mit dem Urin mit einer initialen Halbwertszeit von 13 Stunden (Bereich 9–18 Stunden) und einer geschätzten zweiten Halbwertszeit von 60 Stunden (Bereich 32–94 Stunden) (Jones et al. 2006).

Sechs männliche Probanden im Alter von 20–28 Jahren wurden vier Stunden lang gegen 200 mg **1,3,5-Trimethylbenzol**/m^3^ (ca. 40 ml/m^3^) exponiert. Urinproben wurden vor der Exposition und zweistündlich bis 24 Stunden nach Expositionsbeginn untersucht. Innerhalb von 24 Stunden wurden 3,66 µg (2,16–5,04 µg) und damit 0,0012 % nicht metabolisiertes Trimethylbenzol ausgeschieden mit Halbwertszeiten von 0,45 Stunden (Phase 1) und 6,7 Stunden (Phase 2) (Janasik et al. 2008).

Acht Stunden lang wurden sechs männliche Probanden gegen 20, 60 oder 100 mg **1,3,5-Trimethylbenzol**/m^3^ (ca. 4, 16 oder 20 ml/m^3^) exponiert. Urinproben wurden vor der Exposition und sechs oder acht Stunden nach Expositionsbeginn untersucht. Blut wurde vor der Exposition und 30 Minuten nach Ende der Exposition entnommen. Die Konzentrationen in der Luft korrelierten gut mit der Konzentration im Blut, im Urin und mit der Konzentration des Metaboliten 3,5-Dimethylhippursäure im Urin (Korrelationskoeffizienten 0,929; 0,996 bzw. 0,987) (Janasik et al. 2008).

Bei zwölf Beschäftigten einer Siebdruckerei wurden personengetragene und ortsfest gemessene Luftproben auf die drei Trimethylbenzol-Isomere untersucht. Die Konzentrationen lagen zwischen 0 und 25,3 ml/m^3^ (täglicher zeitgewichteter 8-Stunden-Mittelwert (TWA)). Die Vorschicht-Urinproben aller Beschäftigten enthielten Dimethylhippursäure, was auf die Belastung vom Vortag hinwies. Laut der Autoren würde eine achtstündige TWA-Exposition gegen 25 ml Trimethylbenzol/m^3^ einen Dimethylhippursäure-Wert im Urin von 206 mM/mol Kreatinin nach der Schicht erwarten lassen (Jones et al. 2006).

Drei männliche Probanden im Alter von 23–26 Jahren wurden vier Stunden gegen **Testbenzin** („white spirit“, C9–C12 mit 25,6 % aromatischen Alkylverbindungen) in einer Konzentration von 576 mg/m^3^ (ca. 100 ml/m^3^) exponiert. Die Trimethylbenzol-Konzentration wurde von 2,6 % auf 7,8 % erhöht, um eine bessere analytische Empfindlichkeit zu erreichen. Im Blut stieg die Trimethylbenzol-Konzentration in den 20 Minuten nach Beginn der Exposition schnell an und erreichte nach vier Stunden eine maximale Konzentration von 117 ng Trimethylbenzol/ml Blut. Die Trimethylbenzol-Konzentration im Gehirn wurde mithilfe eines Physiologie-basierten Pharmakokinetik (PBPK)-Modells berechnet und betrug 420 ng/g Gehirn (Hissink et al. 2007).

Fünf Probanden wurden jeweils acht Stunden lang an fünf aufeinander folgenden Tagen gegen 20 ml/m^3^ (100 mg/m^3^) eines Gemischs der drei Trimethylbenzol-Isomeren exponiert. Während der Exposition erfolgte eine körperliche Belastung mit 75 W für jeweils zehn Minuten pro Stunde. Am Ende der einzelnen Expositionstage wurden die Trimethylbenzol-Isomeren im Blut untersucht. Als Summe der drei Isomeren ergaben sich im Blut mittlere Konzentrationen von 607 ± 167 µg Trimethylbenzol/l mit einem 95. Perzentil von 870 µg/l (Kraus et al. 2008).

##### 3.1.1.2 Ratte

Einige Toxikokinetik-Studien mit sehr kurzen Expositionszeiten oder einmaliger Verabreichung sind bereits bei Greim (1998) beschrieben.

Je vier männliche Wistar-Ratten wurden sechs Stunden gegen 25, 100 oder 250 ml **1,2,4-Trimethylbenzol**/m^3^ ganzkörperexponiert. Die Konzentrationen im Blut betrugen am Ende der Exposition 0,94 ± 0,16; 8,32 ± 1,34 bzw. 23,6 ± 1,8 mg/l und die Halbwertszeiten von 1,2,4-Trimethylbenzol im Blut 38 Minuten; 1 Stunde 8 Minuten bzw. 1 Stunden 41 Minuten. Die Halbwertszeiten getrennt nach schneller und langsamer Phase betrugen 10 Minuten, 28 Minuten bzw. 57 Minuten und 3 Stunden 37 Minuten, 20 Minuten (sic!) bzw. 17 Stunden 20 Minuten. Die Bestimmung der Dimethylbenzoesäure-Metaboliten im Urin ergab deutlich höhere Mengen an 3,4-Dimethylbenzoesäure im Vergleich mit 2,5-Dimethylbenzoesäure und 2,4-Dimethylbenzoesäure (Świercz et al. 2002). Eine Halbwertszeit der langsamen Phase von 20 Minuten nach Exposition gegen 100 ml/m^3^ erscheint unplausibel.

Die Ganzkörperexposition männlicher Wistar-Ratten gegen 0, 125, 1250 oder 5000 ml **1,2,4-Trimethylbenzol**/m^3^ führte zu den in Tabelle 2 aufgeführten Konzentrationen in Blut und Gehirn. Nach dreitägiger Exposition gegen 5000 ml/m^3^ wurden niedrigere Gehalte als nach einmaliger achtstündiger Exposition beobachtet (McKee et al. 2010).

**Tab. 2:** 1,2,4-Trimethylbenzol-Konzentrationen in Blut und Gehirn von Ratten nach Exposition (McKee et al. 2010)

**Expositionsdauer**	**1,2,4-Trimethylbenzol Expositions-Konzentration [mg/m^3^]**	**1,2,4-Trimethylbenzol im Blut [ng/ml]**	**1,2,4-Trimethylbenzol im Gehirn [ng/g]**
2 h	0	n. u.	n. u.
	125	211 ± 26	472 ± 42
	1250	4140 ± 411	10 800 ± 624
	5000	37 400 ± 3602	86 667 ± 7049
4 h	0	n. u.	n. u.
	125	198 ± 8	595 ± 58
	1250	5033 ± 33	13 333 ± 601
	5000	57 000 ± 3512	138 333 ± 7265
8 h	0	< 20	< 150
	125	200 ± 16	418 ± 32
	1250	3887 ± 619	10 750 ± 1561
	5000	65 000 ± 3786	160 000 ± 5774
3 × 8 h	0	< 20	< 150
	125	254 ± 31	755 ± 48
	1250	3977 ± 197	11 667 ± 333
	5000	36 167 ± 1590	93 333 ± 2048

h: Stunde; n. u.: nicht untersucht

1,2,4-Trimethylbenzol wurde im Blut, im Gehirn und im perirenalen Fett von Ratten nach jeweils zwölf Stunden Exposition gegen 1000 ml/m^3^ an 14 aufeinanderfolgenden Tagen am 1., 3., 7., 10. und 14. Tag gemessen. Die höchsten Konzentrationen traten am Ende der ersten Exposition auf. Bei den späteren Messungen waren die Konzentrationen von 1,2,4-Trimethylbenzol in den Geweben ca. 50 % geringer. Dies kann mit der Induktion von metabolisierenden Enzymen erklärt werden (Greim 1998).

Je vier männliche Sprague-Dawley-Ratten wurden gegen 100 ml **1,2,4-Trimethylbenzol**/m^3^ (Reinheit > 98 %) zwölf Stunden pro Tag an ein, zwei oder drei aufeinanderfolgenden Tagen ganzkörperexponiert. Am dritten Tag betrug die Trimethylbenzol-Konzentration im Blut 17,1 ± 2,2 µmol/l, im Gehirn 36,5 ± 2,2 µmol/kg, in der Leber 35,4 ± 2,4 µmol/kg, in der Niere 103,6 ± 18,8 µmol/kg und im Fett 1070 ± 93 µmol/kg und wich damit nur gering von den Konzentrationen nach einer ein- oder zweitägigen Exposition ab (Zahlsen et al. 1992).

Je fünf männliche IMP:Wistar-Ratten wurden einmalig sechs Stunden oder vier Wochen, an fünf Tagen pro Woche und sechs Stunden täglich, gegen 0, 25, 100 oder 250 ml **1,3,5-Trimethylbenzol**/m^3^ ganzkörperexponiert. Die Tabellen 3 und 4 geben die 1,3,5-Trimethylbenzol-Konzentrationen und die Konzentrationen des Metaboliten 3,5-Dimethylbenzoesäure in Lunge, Leber, Nieren, Blut und Urin an. In den Nieren reicherten sich Trimethylbenzol und der Metabolit am stärksten an. Nach Beendigung der Exposition wurde 1,3,5-Trimethylbenzol schnell aus dem Blut der Ratten eliminiert. Im Vergleich zur einmaligen Exposition stieg die Konzentration des Metaboliten 3,5-Dimethylbenzoesäure in den Lungen von Ratten nach wiederholter Exposition gegen 100 und 250 ml 1,3,5-Trimethylbenzol/m^3^ und in der Leber bei Exposition gegen 250 ml/m^3^ an, was mit der Induktion von 1,3,5-Trimethylbenzol metabolisierenden Enzymen erklärt werden kann. Die Autoren vermuten, dass der 1,3,5-Trimethylbenzol-Metabolismus in den Lungen der Ratten nach wiederholter Exposition einen statistisch signifikanten Einfluss auf die erhöhte Ausscheidung des Metaboliten 3,5-Dimethylbenzoesäure im Urin hatte. Die Halbwertszeiten von 1,3,5-Trimethylbenzol im Blut nach Beendigung der wiederholten Exposition ergaben für die drei Konzentrationen 23, 8 bzw. 10 Minuten für Phase I (schnell) und 2 Stunden 23 Minuten, 4 Stunden 37 Minuten bzw. 4 Stunden 37 Minuten für die Phase II (Świercz et al. 2006).

**Tab. 3:** 1,3,5-Trimethylbenzol-Konzentrationen nach Ganzkörperexposition in Geweben und venösem Blut von Ratten (Świercz et al. 2006)

**Zeit**	**Konzentration ml/m^3^**	**Leber** **µg/g**	**Lunge** **µg/g**	**Nieren** **µg/g**	**Blut** **µg/ml**
nach 6 h Exposition	25	0,30 ± 0,07^a)^	0,31 ± 0,12	4,49 ± 1,93	0,31 ± 0,12
	100	3,09 ± 0,50	2,87 ± 0,57	13,32 ± 2,58	3,06 ± 0,651
	250	17,00 ± 6,08	17,36 ± 5,56	31,80 ± 9,44	13,36 ± 1,54
nach 4 Wo Exposition	25	0,22 ± 0,01	0,42 ± 0,12	1,73 ± 0,30*	0,31 ± 0,08
	100	3,01 ± 0,58	1,99 ± 0,75	15,61 ± 2,14	2,30 ± 0,52
	250	12,98 ± 4,16	11,20 ± 3,61	35,97 ± 8,53	7,55 ± 1,43**

* p ≤ 0,05;

** p ≤ 0,01 im Vergleich zur einmaligen Exposition (Students t-Test)

a) Mittelwerte ± Standardabweichung

h: Stunde; Wo: Woche

**Tab. 4:** 3,5-DMBA-Konzentrationen nach Ganzkörperexposition gegen 1,3,5-Trimethylbenzol in Geweben und Urin von Ratten (Świercz et al. 2006)

**Zeit**	**Konzentration ml/m^3^**	**3,5-DMBA-Konzentration**			
		**Leber** **µg/g**	**Lunge** **µg/g**	**Niere** **µg/g**	**Urin** **mg/18 h**
nach 6 h Exposition	25	12,62 ± 1,62^a)^	2,87 ± 0,55	8,77 ± 0,99	0,52 ± 0,03
	100	26,05 ± 2,77	5,50 ± 0,55	27,01 ± 9,86	3,66 ± 0,57
	250	36,92 ± 1,61	13,39 ± 1,90	60,91 ± 19,78	10,99 ± 3,90
nach 4 Wo Exposition	25	6,52 ± 0,67**	3,69 ± 1,21	11,06 ± 4,33	0,83 ± 0,15*
	100	21,67 ± 3,14**	8,90 ± 0,98**	31,03 ± 18,56	4,36 ± 0,86
	250	53,07 ± 5,41**	19,79 ± 2,70**	82,10 ± 14,48	11,92 ± 3,05

* p ≤ 0,05;

** p ≤ 0,01 im Vergleich zur einmaligen Exposition (Students t-Test)

a) Mittelwerte ± Standardabweichung

DMBA: Dimethylbenzoesäure; h: Stunde; Wo: Woche

Die einmalige sechsstündige oder vierwöchige Ganzkörperexposition von je vier männlichen Wistar-Ratten gegen 0, 25, 100 oder 250 ml **1,2,4-Trimethylbenzol**/m^3^, an fünf Tagen pro Woche und sechs Stunden täglich, führte zu niedrigeren Konzentrationen im Blut verglichen mit denen in Gehirn, Leber und Lunge. Die 1,2,4-Trimethylbenzol-Konzentrationen in der Leber lagen nach einmaliger Exposition gegen 100 oder 250 ml/m^3^ mit 7,13 ± 1,31 bzw. 28,18 ± 5,34 mg/kg höher als nach vierwöchiger Exposition (3,00 ± 0,49 bzw. 22,47 ± 4,10 mg/kg). In Lunge (4,14 ± 0,54 bzw. 3,74 ± 0,82 mg/kg und 18,90 ± 3,72 bzw. 22,47 ± 4,10 mg/kg) und Gehirn (2,92 ± 0,73 bzw. 2,82 ± 0,40 und 18,34 ± 1,92 bzw. 18,63 ± 4,27 mg/kg) traten diese Unterschiede nicht auf. Nach sechsstündiger Exposition gegen 25, 100 oder 250 ml/m^3^ betrugen die Konzentrationen im Blut 0,31 ± 0,12; 1,24 ± 0,41 bzw. 7,76 ± 1,64 mg/l, nach vierwöchiger Exposition 0,33 ± 0,11; 1,54 ± 0,32 bzw. 7,52 ± 2,11 mg/l und die Halbwertszeiten von 1,2,4-Trimethylbenzol im Blut nach Beendigung der wiederholten Exposition ergaben 9, 32 bzw. 68 Minuten für die Phase I (schnell) und 2 Stunden 53 Minuten, 5 Stunden 47 Minuten bzw. 9 Stunden 54 Minuten für die Phase II. Im Gehirn reicherten sich nach sechsstündiger Exposition 0,49 ± 0,06; 2,92 ± 0,73 bzw. 18,34 ± 1,92 mg 1,2,4-Trimethylbenzol/kg an und nach vierwöchiger Exposition 0,45 ± 0,05; 2,82 ± 0,40 bzw. 18,63 ± 4,27 mg 1,2,4-Trimethylbenzol/kg (siehe auch Abschnitt 5.2.1; Świercz et al. 2003).

Je fünf männliche Wistar-Ratten wurden einmalig sechs Stunden oder vier Wochen lang, an fünf Tagen pro Woche und sechs Stunden täglich, gegen 0, 25, 100 oder 250 ml **1,2,3-Trimethylbenzol**/m^3^ (Reinheit 90 %) ganzkörperexponiert. Die Konzentrationen im Blut betrugen nach sechsstündiger Exposition 0,76 ± 0,09; 3,82 ± 0,94 bzw. 10,75 ± 1,3 mg/l und die Halbwertszeiten im Blut 38 Minuten; 1 Stunde 8 Minuten bzw. 1 Stunde 41 Minuten. Die Halbwertszeiten getrennt nach schneller und langsamer Phase beliefen sich auf 14 Minuten, 19 Minuten bzw. 32 Minuten und 3 Stunden 4 Minuten, 3 Stunden 42 Minuten bzw. 5 Stunden 20 Minuten. Nach vierwöchiger Exposition betrugen die 1,2,3-Trimethylbenzol-Konzentrationen im Blut 0,58 ± 0,08; 3,14 ± 0,61 bzw. 6,87 ± 1,05 mg/l und die Halbwertszeiten in der Phase I 2 Minuten, 8 Minuten bzw. 13 Minuten und in Phase II 5 Stunden 52 Minuten, 4 Stunden 34 Minuten bzw. 7 Stunden 58 Minuten. Der Metabolit 2,3-DMBA wurde in Leber, Lunge und Nieren bestimmt. Die Konzentrationen lagen nach vierwöchiger Exposition gegen 100 oder 250 ml/m^3^ in Leber und Nieren statistisch signifikant niedriger als nach sechsstündiger Exposition. In der Lunge konnte der Metabolit nur bei den Tieren der höchsten Konzentrationsgruppe mit 3,23 ± 0,56 bzw. 2,82 ± 0,44 µg/g detektiert werden. Die Nachweisgrenze lag bei 0,25 µg/g Gewebe (Nassgewicht). Weder in der Leber noch in der Lunge oder den Nieren der Ratten wurde 2,6-DMBA nachgewiesen (Świercz et al. 2016).

Je drei bis vier männliche Wistar-Ratten wurden zwei, vier oder acht Stunden lang gegen 0, 600, 2400 oder 4800 mg **Testbenzin** („white spirit“, C9–C12 mit 25,6 % aromatischen Alkylverbindungen)/m^3^ (ca. 0, 102, 410 oder 820 ml/m^3^) exponiert. Die Trimethylbenzol-Konzentration wurde von 2,6 % auf 7,8 % erhöht, um eine bessere analytische Empfindlichkeit zu erreichen. Die Bestimmung der Trimethylbenzol-Konzentrationen in Blut und Gehirn ergab, dass bei den Tieren der höchsten Konzentrationsgruppe kein Fließgleichgewicht eintrat, was auf eine Sättigung des Metabolismus oder eine Diffusionsbegrenzung hinweist (Hissink et al. 2007).

#### 3.1.2 Orale Aufnahme

Je vier männliche WAG/Rij-Ratten erhielten einmalig oral, vermutlich per Schlundsonde, 0,008; 0,016 oder 0,032 mol/kg KG (ca. 0, 960, 1923 oder 3846 mg/kg KG) von einem der drei Isomeren **1,2,4-Trimethylbenzol**, Reinheit 97 %, **1,2,3-Trimethylbenzol**, Reinheit 90–95 %, oder **1,3,5-Trimethylbenzol**, Reinheit 99 %, in Olivenöl gelöst. Die Untersuchung des Blutes in zehnminütigen Abständen ergab 20 Minuten nach der Gabe einen konzentrationsabhängigen Anstieg der drei Isomere. Nur 1,2,4-Trimethylbenzol war bereits zehn Minuten nach der Gabe nachweisbar. Die Konzentration im Blut nahm bei 1,2,4-Trimethylbenzol am schnellsten ab, während 1,3,5-Trimethylbenzol deutlich länger im Blut nachweisbar war. Damit verlief bei den drei Isomeren die Aufnahme und Abnahme und damit der Übergang ins Gewebe unterschiedlich (Tomas et al. 1999 b).

#### 3.1.3 Dermale Aufnahme

##### 3.1.3.1 In-vitro-Studien mit humaner Haut

In einer In-vitro-Studie wurde die Resorption von unverdünntem **1,2,4-Trimethylbenzol** sowie von 50- und 10%igen Lösungen in Ethanol in statischen Franz-Zellen (Diffusionsbereich: 0,64 cm^2^, Kapazität des Rezeptor-Flüssigkeits-Kompartiments: 5,1 ml) mit humaner Haut (Dicke: 0,9 mm) eines Donors gemessen. Die Rezeptorlösung bestand aus Ethanol. Appliziert wurden 128 µl/0,64 cm^2^ (200 µl/cm^2^) okklusiv über einen Zeitraum von acht Stunden. Die Probenahmen erfolgten 0,5; 1; 2; 4 und 8 Stunden nach Applikation. Die durch die Haut penetrierten Mengen lagen für unverdünntes **1,2,4-Trimethylbenzol** bei 64 µg, für die 50 % bzw. 10 % verdünnte Lösung bei 77 µg und 6 µg (Korinth et al. 2003). Für unverdünntes **1,2,4-Trimethylbenzol** und den Zeitraum von 0,5–8 Stunden kann ein Flux von 12,5 µg/cm^2^ und Stunde berechnet werden (64 µg/0,64 cm^2^/8 h). Für die 50 % bzw. 10 % verdünnten Lösungen ergeben sich Flux-Werte von 15,04 µg/cm^2^ und Stunde bzw. 1,17 µg/cm^2^ und Stunde. Aus dem Flux ergibt sich unter Standardbedingungen (eine Stunde Exposition von beiden Händen und Unterarmen, Penetrationsfläche von 2000 cm^2^) eine Aufnahme von 25 mg (unverdünnt), 30 mg (50%ige Lösung) sowie 2,34 mg (10%ige Lösung). Anzumerken ist, dass die Verwendung von Ethanol als Rezeptorflüssigkeit einen Worst Case darstellt. Weiterhin schreiben die Autoren, dass das Fließgleichgewicht nicht erreicht wurde.

##### 3.1.3.2 In-vivo- und In-vitro-Studien am Tier

In einer In-vitro-Studie mit einem Durchfluss-Diffusions-Zellsystem mit Schweinehaut (Dicke: 0,5 mm, Fläche 0,64 cm^2^) wurde die Hautresorption von Trimethylbenzol (k. A. zu den Isomeren) untersucht. Es wurden 355,6 µg Trimethylbenzol, gelöst in 20 µl eines Wasser-Ethanol-Gemischs (Verhältnis 50:50), auf die Haut gegeben. Die Expositionsdauer betrug einen Tag sowie vier Tage mit wiederholter täglicher Exposition. Die eintägige Exposition ergab einen Flux im Fließgleichgewicht von 1,01 ± 0,14 µg/cm^2^ und Stunde, die wiederholte Exposition von 0,49 ± 0,04 µg/cm^2^ und Stunde. Die Permeabilität ist mit 0,056 ± 0,008 cm/h × 1000 bzw. 0,028 ± 0,002 cm/h × 1000 angegeben (Muhammad et al. 2005). Aus den Fluxen nach 24 Stunden und vier Tagen ergeben sich unter Standardbedingungen (eine Stunde Exposition von beiden Händen und Unterarmen, Penetrationsfläche von 2000 cm^2^) Aufnahmen von 2,02 mg bzw. 0,98 mg.

In einem Experiment mit einer statischen Diffusionszelle wurde auf 4,9 cm^2^ Haut von F344-Ratten „JP-8“ aufgetragen, ein Treibstoff, der 1 % Trimethylbenzol (k. A. zu den Isomeren) enthält. Das Rezeptorkompartiment wurde mit 6 % „Volpo 20“, einem Lösemittel, in physiologischer Kochsalzlösung befüllt. Die Expositionszeit betrug vier Stunden. Für Trimethylbenzol wurde ein Flux von 1,25 ± 0,5 µg/cm^2^/h angegeben (McDougal et al. 2000).

Sprague-Dawley-Ratten wurde für zwei sowie 24 Stunden okklusiv Kerosin aufgetragen, welches 80 % gesättigte Kohlenwasserstoffe (Naphthene (Cycloalkane) und aliphatische Kohlenwasserstoffe) und 20 % Aromaten (u. a. Trimethylbenzol) enthielt. In der Rattenhaut wurden die Isomere **1,3,5-Trimethylbenzol**, **1,2,4-Trimethylbenzol** und **1,2,3-Trimethylbenzol** analytisch bestimmt, wobei die angegebenen Werte die Summe der drei Isomere darstellen. Bei einer Auftragung von 1 ml Kerosin/cm^2^ oder 0,25 ml Kerosin/cm^2^ wurde in der Rattenhaut ein Trimethylbenzol-Gehalt von 4,56 ± 0,86 µg/g bzw. 4,61 ± 0,67 µg/g bestimmt. In einem weiteren Versuch wurde in vitro mittels einer Franz-Diffusionszelle (Fläche 1,76 cm^2^) für 24 Stunden die Hautresorption des Kerosins untersucht. In der Rezeptorflüssigkeit ließen sich bei Auftragung von 17,05 µl Kerosin/cm^2^ und 68,2 µl Kerosin/cm^2^ Trimethylbenzol-Gehalte von 0,09 ± 0,01 µg/g bzw. 0,33 ± 0,06 µg/g nachweisen (Tsujino et al. 2003). Aus den In-vivo-Untersuchungen lässt sich kein Flux und damit keine dermale Aufnahmemenge berechnen. Aus der In-vitro-Untersuchung kann bei einem Trimethylbenzol-Gehalt der Rezeptorflüssigkeit von 0,09 ± 0,01 µg/g, der Auftragungsfläche von 1,76 cm^2^ Haut sowie der entnommenen Rezeptorflüssigkeit von 2 ml (Annahme: 2 ml = 2 g) eine penetrierte Trimethylbenzol-Gesamtmenge von 0,10 µg/cm^2^ in 24 Stunden abgeschätzt werden. Daraus errechnet sich ein Flux von 0,0042 µg/cm^2^ und Stunde sowie eine dermale Aufnahme unter Standardbedingungen (eine Stunde Exposition von beiden Händen und Unterarmen, Penetrationsfläche von 2000 cm^2^) von 0,0085 mg. Analog kann bei einem Trimethylbenzol-Gehalt der Rezeptorflüssigkeit von 0,33 ± 0,06 µg/g ein Flux von 0,0156 µg/cm^2^ und Stunde sowie eine dermale Aufnahme unter Standardbedingungen (siehe oben) von 0,0312 mg abgeschätzt werden.

##### 3.1.3.3 Fazit dermale Aufnahme

Die verfügbare Studie (Korinth et al. 2003) zur dermalen Aufnahme mit humaner Haut in vitro zeigt für **1,2,4-Tri­methylbenzol** unverdünnt und in zwei verschiedenen Verdünnungen (10 % und 50 %) bzw. Trimethylbenzol (k. A. zu den Isomeren) geringe Flux-Werte von maximal 15,01 µg/cm^2^ und Stunde. Dies entspricht einer dermalen Aufnahme (einstündige Exposition von beiden Händen und Unterarmen mit Penetrationsfläche von ca. 2000 cm^2^) von 30 mg. Bei diesen Studien wurde Ethanol in der Rezeptorflüssigkeit eingesetzt, was einen Worst Case darstellt, da sich Trimethylbenzol darin besser als in Wasser löst.

Weiterhin gibt es drei Untersuchungen zur dermalen Aufnahme an Schweine- bzw. Rattenhaut (McDougal et al. 2000; Muhammad et al. 2005; Tsujino et al. 2003), wobei sich Flux-Werte von 0,0042 µg/cm^2^ und Stunde bis 1,25 µg/cm^2^ und Stunde ergeben. Anhand des höchsten Flux-Wertes von 1,25 µg/cm^2^ und Stunde kann somit eine maximale Aufnahmemenge unter Standardbedingungen (2000 cm^2^, 1 h) von 2,5 mg berechnet werden. Auch wenn die Rattenhaut aufgrund einer zwei- bis dreifach höheren Permeabilität im Vergleich zur menschlichen Haut (McDougal et al. 2000) nicht zur Berechnung der humanen Hautresorption herangezogen werden kann, bestätigen die Studien eine sehr geringe Hautresorption.

Die Gesamtschau der Daten zur dermalen Aufnahme zeigt anhand von Untersuchungen mit humaner Haut und Tierhaut eine geringe Resorption, wobei die Studie von Korinth et al. (2003) mit 30 mg 1,2,4-Trimethylbenzol die höchste Resorption ergibt und deshalb für die Bewertung der Hautresorption herangezogen wird.

### 3.2 Metabolismus

#### 3.2.1 Mensch

Der Hauptmetabolisierungspfad der Trimethylbenzole ist die Hydroxylierung einer der Methylgruppen mit nachfolgender Oxidation zur entsprechenden DMBA. Diese kann nach Konjugation mit Glycin als Dimethylhippursäure im Urin als ein Hauptmetabolit ausgeschieden werden (Kraus et al. 2008).

#### 3.2.2 Ratte

Die **1,2,4-Trimethylbenzol**-Konzentration in der Leber war nach wiederholter Exposition gegen 100 oder 250 ml/m^3^ bei Ratten statistisch signifikant niedriger als nach einmaliger Exposition. Dies könnte auf die Induktion von Enzymen in der Leber hinweisen (Świercz et al. 2003).

### 3.3 Fazit

Die Aufnahme von Trimethylbenzol führt zur Induktion metabolisierender Enzyme. Nach Exposition gegen 250 ml/m^3^ kam es zu einer Überlastung des Metabolismus bei Ratten. Inhalationsstudien am Menschen und mit Tieren belegen eine ähnliche Eliminationskinetik und einen ähnlichen Metabolismus der drei Trimethylbenzol-Isomere, der zu DMBA-Metaboliten führt.

## 4 Erfahrungen beim Menschen

### 4.1 Einmalige Exposition

Zehn männliche Probanden wurden zwei Stunden lang in einer Expositionskammer gegen 25 ml **1,2,4-Trimethylbenzol**/m^3^, 25 ml **1,2,3-Trimethylbenzol**/m^3^, 25 ml **1,3,5-Trimethylbenzol**/m^3^ oder gegen 2 ml 1,2,4-Trimethylbenzol/m^3^ exponiert und verrichteten dabei konstant 50 W Arbeit auf einem Fahrradergometer. Die Probanden wurden per Fragebogen nach Irritationswirkungen oder ZNS-Effekten befragt. Es wurden keine signifikanten Wirkungen angegeben (Greim 1998).

Neun männliche Probanden wurden zwei Stunden lang in einer Expositionskammer gegen 11 mg **1,2,4-Trimethylbenzol**/m^3^ (2 ml/m^3^) oder 11 mg **1,2,4-Trimethylbenzol/m^3^ in 300 mg Testbenzin** („white spirit“)/m^3^ unter Belastung mit 50 W (Fahrradergometer) exponiert. Es zeigte sich während und bis zu vier Stunden nach der Exposition kein Einfluss auf Herzfrequenz oder Atmung. Die Probanden gaben in einem Fragebogen an, dass keine Reizwirkung und keine weiteren Beeinträchtigungen durch Müdigkeit, Kopfschmerzen, Übelkeit, Schwindel, Atembeschwerden oder Geruchsbelästigung auftraten (Järnberg et al. 1998).

In einer Expositionskammer wurden vier Probanden vier Stunden lang gegen 25 ml **1,3,5-Trimethylbenzol**/m^3^ exponiert. Die Ermittlung der Reizwirkung erfolgte mit einem Fragebogen. Die Geruchswahrnehmung von 1,3,5-Trimethylbenzol nahm mit längerer Expositionszeit ab. Die Probanden gaben minimale („very little“) sensorische Reizwirkungen an (k. w. A.; Jones et al. 2006).

### 4.2 Wiederholte Exposition

Studien nach wiederholter Exposition wurden bereits im Nachtrag aus dem Jahr 1998 (Greim 1998) beschrieben, bei denen jedoch immer Mischexposition vorlag. Im Folgenden werden Studien, die nach 1998 publiziert worden sind, ausführlich beschrieben.

In einer Probandenstudie wurden zwölf Männer gegen 57 mg **Testbenzin** („white spirit“, C9–C12 mit 21,3 % aromatischen Alkylverbindungen)/m^3^ zweimal mit einem Abstand von einer Woche jeweils vier Stunden exponiert. Die gleiche Gruppe an Probanden wurde sieben Tage später gegen 570 mg Testbenzin/m^3^ unter den gleichen Bedingungen exponiert. Blutproben wurden zehn Minuten vor Ende der Expositionszeit genommen und die Konzentrationen von 1,2,4-Trimethylbenzol und *n*-Decan bestimmt. Nach Exposition gegen 570 mg/m^3^ betrug die mittlere Blutkonzentration von 1,2,4-Trimethylbenzol 58,2 ng/ml (46–77 ng/ml). Bei diesen höher exponierten Probanden trat leicht erhöht Müdigkeit auf. Es wurde eine statistisch signifikante Beziehung zwischen abnehmender Leistung im Pattern Comparison Test und 1,2,4-Trimethylbenzol-Konzentrationen im Blut beobachtet. Eine statistisch signifikant längere Latenzzeit in einem Aufmerksamkeits-Test (Switching Attention Test) korrelierte ebenfalls mit höheren 1,2,4-Trimethylbenzol-Konzentrationen im Blut. In weiteren durchgeführten Tests (Auge-Hand-Koordinationstest, Fingertipp-Test, Verbaler Gedächtnistest, Test zur räumlichen Gedächtnisspanne, Test zur Gedächtnisspanne, einfacher Reaktionszeittest, Color Word Vigilance Test, Profile of Mood States Questionnaire, Zahlen-Symbol-Test) wurden keine Effekte beobachtet (Lammers et al. 2007). Die genauen Trimethylbenzol-Konzentrationen im Blut von drei Probanden sind nur für die 570 mg/m^3^-Gruppe in einem Diagramm dargestellt. Es kann nicht ausgeschlossen werden, dass die beobachteten Effekte durch weitere Substanzen des Gemisches verstärkt wurden.

Bei zwölf Beschäftigten einer Siebdruckerei wurden personengetragen und ortsfest gesammelte Luftproben auf die drei Trimethylbenzol-Isomeren untersucht. Die Konzentrationen lagen zwischen 0 und 25,3 ml/m^3^ (täglicher zeitgewichteter 8-Stunden-Mittelwert) (Jones et al. 2006). Es werden keine Angaben zu Reizeffekten oder systemischen Effekten der Exponierten gemacht.

Die Konzentrationen der drei Trimethylbenzol-Isomere sowie von Xylol, Toluol, Ethyltoluol, Styrol und *n*-Propylbenzol in einem **Alkylbenzol-Gemisch**, gegen das Beschäftigte in der Farbenindustrie exponiert waren, wurden ermittelt (personengetragene Messung). Trimethylbenzole machten etwa 42 % und Xylol ca. 20 % im Alkylbenzol-Gemisch aus. Es wird eine berechnete kombinierte Gesamtexposition (Summe der Quotienten aus den Konzentrationen der einzelnen Verbindungen durch die jeweiligen Expositionsgrenzwerte) von 0,94–3,73 mit Xylol (Bereich 0,3–12,38) und ohne Xylol 0,83–1,3 (Bereich 0,4–2,9) (geometrische Mittelwerte) angegeben. Die Autoren weisen darauf hin, dass die Xylol-Konzentration leicht über dem in Polen gültigen Grenzwert von 100 mg/m^3^ liegt. Von 95 Beschäftigten wurden 34 Personen aufgrund von Mittelohrerkrankungen, zurückliegenden Ohroperationen, Kopfverletzungen, Einnahme ototoxischer Medikamente, Diabetes, Bluthochdruck, neurologischen Krankheiten, Alkoholmissbrauch oder Lärmbelastung in der Vergangenheit nicht in die Studie aufgenommen. Die Beschäftigungsdauer lag zwischen zwei und 34 Jahren. Verglichen wurde mit 40 Kontrollpersonen, die in der gleichen Firma in Verwaltung und Transport beschäftigt waren. Die 61 männlichen Beschäftigten wurden gruppiert nach der berechneten kumulativen Dosis von < 10; 10–20 und > 20. Die entsprechenden Mittelwerte betrugen 5,46 ± 2,48, 20 Personen, mittleres Alter 35 Jahre; 15,18 ± 3,43, 23 Personen, mittleres Alter 36,3 Jahre und 41,88 ± 14,36, 18 Personen, mittleres Alter 41,4 Jahre. Die berechnete kombinierte Gesamtexposition betrug in der niedrigsten Expositionsgruppe 0,94 ± 0,75 und liegt damit nur bei den am niedrigsten Exponierten unter der Hygienenorm von 1. Von 61 untersuchten Beschäftigten traten bei 42 % Hörverluste („Pure-Tone Audiogram“) auf, ab einer kumulativen Dosis > 10 waren Hörverluste häufiger (verminderte Wahrnehmung ab einer Lautstärke > 16 dB), steigerten sich jedoch nicht in der Gruppe der am stärksten belasteten. Gleichgewichtssinn-Störungen traten bei 47,5 % und Schwindel bei 26,1 % der exponierten Beschäftigten auf. Bei 5 % der Kontrollpersonen wurden Hörverluste und Gleichgewichtssinn-Störungen nachgewiesen. Es erfolgte keine Aussage zum Ausmaß des Schwindels der Kontrollpersonen. Nach Aussage der Autoren werden alle Grenzwerte für die einzelnen Substanzen eingehalten, außer manchmal für Xylol. Trotzdem traten bei 42 % der Exponierten Hörverluste auf, was auf eine Kombinationswirkung der Lösungsmittel hinweist (Sułkowski et al. 2002). Da das Expositionsgemisch unterschiedliche Lösungsmittel enthielt, die, hier insbesondere Xylol, ototoxisch wirken, und Konzentrationsangaben fehlen, kann diese Studie nicht zur Bewertung der ototoxischen Wirkung der Trimethylbenzol-Isomere herangezogen werden.

Bei gegen ein Lösungsmittelgemisch-exponierten Beschäftigten war die Leistung bei Gedächtnisaufgaben und die Aktivität in den Gehirnregionen, die für Aufmerksamkeit und Arbeitsgedächtnis (anteriorer cingulärer, präfrontaler und parietaler Cortex) verantwortlich sind, statistisch signifikant vermindert (Tang et al. 2011). Es fehlt eine Angabe über die Trimethylbenzol-Konzentration in dem Lösungsmittelgemisch. Die Studie wird nicht zur Bewertung herangezogen.

Die durch einen Fragebogen ermittelten neurologischen Einschränkungen von Werftlackierern durch Lösungsmittelexposition (Lee et al. 2005) werden nicht zur Bewertung herangezogen, da das Lösungsmittelgemisch nur einen geringen Prozentsatz von ca. 5 % Trimethylbenzol (3,71 ml Trimethylbenzol/m^3^) enthielt und die Effekte daher nicht auf Trimethylbenzol zurückgeführt werden können.

### 4.3 Wirkung auf Haut und Schleimhäute

Die okklusive Auftragung von flüssigem **1,3,5-Trimethylbenzol** (2 cm^2^ großes Gazepflaster) auf die dorsale Haut der Hand eines männlichen Probanden führte zu einer raschen Entwicklung von leichtem Juckreiz, Erythem und Ödem an der Behandlungsstelle (innerhalb von 30 Minuten). Das schnelle Einsetzen der Reizung bei dieser Person deutet eher auf eine primäre Reizwirkung und nicht auf eine immunologisch vermittelte Reaktion hin (Jones et al. 2006).

### 4.4 Allergene Wirkung

#### 4.4.1 Hautsensibilisierende Wirkung

In einem Firmenbericht aus dem Jahr 1983 wird über einen humanen Epikutantest mit **1,2,4-Trimethylbenzol** berichtet, wobei neben starken irritativen Effekten auch ausgeprägte photoallergische Effekte beobachtet wurden (Dupont 1983, liegt nicht als Originalstudie vor, bei ECHA (2023) sekundär zitiert). Es fehlen jegliche weitere Angaben zur Studie. Eine photosensibilisierende Wirkung erscheint aufgrund der chemischen Struktur nicht plausibel und wurde seitdem nicht reproduziert. Auch von strukturanalogen Verbindungen (beispielsweise Toluol, Xylol, Ethylbenzol, Triethylbenzol, Styrol) ist keine photosensibilisierende Wirkung bekannt.

#### 4.4.2 Atemwegssensibilisierende Wirkung

Es liegen keine gezielten Untersuchungen zur atemwegssensibilisierenden Wirkung vor.

In einer Bevölkerungsstudie zur Hintergrundbelastung flüchtiger organischer Verbindungen (u. a. 1,2,4-Trimethylbenzol) wurde ein Zusammenhang zwischen der Raumluftkonzentration in Wohninnenräumen und Asthma und Rhinitis bei Erwachsenen gefunden. Flüchtige organische Verbindungen werden im Haushalt von Baustoffen, Farben, Möbeln, Teppichen, TV-Geräten etc. emittiert, wobei 1,2,4-Trimethylbenzol beispielsweise in Oberflächenbeschichtungen, Klebstoffen und Gummi vorkommt. In 490 Wohnungen wurde als Hintergrundbelastung durch **1,2,4-Trimethylbenzol** ein Median von 4,0 µg/m^3^ bestimmt, wobei in den einzelnen Wohnungen minimale Werte unterhalb des Detektionslimits (0,03 µg/m^3^) bis zu einer maximalen Konzentration von 111,7 µg/m^3^ gemessen worden waren. Von 1012 Personen, die in den untersuchten Wohnungen lebten, gaben 8,6 % an, Asthmasymptome zu haben und 38,3 % Rhinitis. Es wurde festgestellt, dass die Exposition gegen 1,2,4-Trimethylbenzol statistisch signifikant mit Asthma verbunden ist (Billionnet et al. 2011), es wurde jedoch keine Untersuchung durchgeführt, um festzustellen, ob eine Sensibilisierung gegen 1,2,4-Trimethylbenzol vorliegt. Rückschlüsse auf eine mögliche sensibilisierende Wirkung von 1,2,4-Trimethylbenzol können daher nicht gezogen werden.

Daten mit beruflicher Exposition liegen nicht vor.

### 4.5 Reproduktionstoxizität

Hierzu liegen keine Daten vor.

### 4.6 Genotoxizität

Hierzu liegen keine Daten vor.

### 4.7 Kanzerogenität

Hierzu liegen keine Daten vor.

## 5 Tierexperimentelle Befunde und In-vitro-Untersuchungen

### 5.1 Akute Toxizität

#### 5.1.1 Inhalative Aufnahme

Die LC_50_-Werte waren bei Ratten nach vierstündiger Exposition 18 000 mg **1,2,4-Trimethylbenzol**/m^3^ (ca. 3600 ml/m^3^) und 24 000 mg **1,3,5-Trimethylbenzol**/m^3^ (ca. 4800 ml/m^3^) (Firth 2008).

Die LC_50_ betrug bei einem **C9–C10-Gemisch** (Destillat aus Naphtha bei 155–200 °C) 2770 ml/m^3^ für die Ratte nach sieben Stunden und 1900 ml/m^3^ nach 3,7 Stunden für die Maus (Nau et al. 1966).

Nach vierstündiger Exposition von Ratten gegen 250–2000 ml **Trimethylbenzol**/m^3^ verursachte jedes der drei Isomere eine konzentrationsabhängige Verminderung der Leistung im Rotarod-Test, die EC_50_ lag für 1,2,4-Trimethylbenzol bei 954 ml/m^3^, für 1,3,5-Trimethylbenzol bei 963 ml/m^3^ und für 1,2,3-Trimethylbenzol bei 768 ml/m^3^. Im Hot-Plate-Test war bei allen drei Isomeren die Latenzzeit verlängert (Greim 1998). Nach Angabe der Autoren wurde der Hot-Plate-Test unmittelbar nach Beendigung der Exposition durchgeführt. Auffallend war jedoch, dass die beiden Kontrollgruppen (akut und subchronisch, siehe Abschnitt 5.2.2) Unterschiede in der Latenzzeit um ca. 50 % aufwiesen. Die Autoren erklärten dies durch die verschiedenen Generationen F2 bzw. F7 mit denen die Tests durchgeführt wurden (Greim 1998).

Eine vierstündige Exposition von je zehn männlichen BALB/c-Mäusen gegen 0, 4665, 5497, 6635 oder 9622 mg **Farbasol** (96 % C9-Alkylbenzol, davon 46 % Trimethylbenzol, 40 % Ethyltoluol)/m^3^ führte im direkt anschließend durchgeführten Rotarod-Test zu Fehlern bei 0/10, 4/10, 5/10, 8/10 bzw. 10/10 Tieren. Die Abnahme der Schmerzsensibilität im Hot-Plate-Test betrug 0, 14,4 %; 20,8 %; 53,8 % bzw. 100 % (Korsak et al. 1999).

Eine sechsstündige Ganzkörperexposition von je fünf männlichen Wistar-Ratten gegen 0, 25, 100 oder 250 ml **1,2,3-Trimethylbenzol**/m^3^ (Reinheit > 90 %), **1,2,4-Trimethylbenzol** (Reinheit > 97 %) oder **1,3,5-Trimethylbenzol** (Reinheit > 99 %) verursachte keine veränderte Körpergewichtszunahme und zeigte keinen Einfluss auf das relative Leber-, Nieren- und Lungengewicht. Im Vergleich mit einer vierwöchigen Exposition wurden im Blut und in Geweben statistisch signifikant höhere Konzentrationen beobachtet. Die Autoren führen das auf eine geringere Retention der Chemikalie in den Lungen der Ratten nach längerer Exposition zurück (Świercz et al. 2002, 2006, 2016).

#### 5.1.2 Orale Aufnahme

Die LD_50_-Werte betrugen 3400 bzw. 6000 mg **1,2,4-Trimethylbenzol**/kg KG (keine Angabe der Spezies) und 3450 bzw. 3500 mg **1,2,3-Trimethylbenzol** bei Ratten (Greim 1998).

Nach einmaliger oraler Schlundsondengabe an je fünf weibliche und männliche Albino-Ratten von 3–6,3 g **Alkylbenzol-Gemisch**/kg KG mit einer Beobachtungszeit von 14 Tagen wurde eine LD_50_ von 5,1 g/kg KG ermittelt (FMC Corp 1984). Neben der Angabe, dass das Gemisch die drei Isomere des Trimethylbenzols und des Ethyltoluols enthielt, erfolgte keine Information zu den Konzentrationsverhältnissen.

Nach einer einmaligen Schlundsondengabe von 0; 3,1; 5,0; 7,12 oder 10,14 g **1,2,4-Trimethylbenzol**/kg KG an je zehn männliche Wistar-Ratten wurde nach 14-tägiger Beobachtungsdauer eine LD_50_ von 6,0 g/kg KG ermittelt. Bei allen behandelten Tieren traten Lethargie und Ptosis auf (MB Research Laboratories 1982 a).

Je fünf männliche oder weibliche CD-Ratten erhielten 0; 1,47; 2,15; 3,16; 4,64; 6,81 oder 11,0 g **1,2,4-Trimethylbenzol**/kg KG einmalig per Schlundsonde. Aus den Todesfällen innerhalb von 14 Tagen wurden LD_50_-Werte von 3,55 bzw. 3,28 g/kg KG ermittelt. Ab der niedrigsten Dosis wurde Inaktivität beobachtet und mit steigender Dosis erhöhter Speichelfluss, Piloarrektion, unkoordinierte Bewegungen, gewölbter Rücken und Erschöpfung (Litton Bionetics Inc 1976).

Je zehn männliche WAG/Rij-Ratten erhielten oral, vermutlich per Schlundsonde, 0; 0,002; 0,008; 0,016 oder 0,032 mol/kg KG (ca. 0, 240, 960, 1923 oder 3846 mg/kg KG) von einem der drei Isomere **1,2,4-Trimethylbenzol**, Reinheit 97 %, **1,3,5-Trimethylbenzol**, Reinheit 99 %, oder **1,2,3-Trimethylbenzol**, Reinheit 90–95 %, in Olivenöl gelöst. Die zehn Kontrolltiere erhielten Olivenöl. Die lokomotorische Aktivität (Open-Field-Test) war bei den behandelten Ratten bis zu einer Dosis von 0,016 mol/kg KG geringfügig eingeschränkt, und die Tiere zeigten eine gering gesteigerte Piloarrektion. Bei den Tieren der höchsten Dosisgruppe wurde 15 Minuten nach der Gabe eine gesteigerte lokomotorische Aktivität und ein verändertes Gangbild mit Parese der Hinterextremitäten beobachtet. Zudem traten eine erhöhte Atemfrequenz (Tachypnoe) und Tremor auf. Verstärkte blutige Sekretion der oberen Atemwege wurde hauptsächlich bei den gegen 1,2,4- und 1,2,3-Trimethylbenzol exponierten Tieren beobachtet. 24 Stunden nach der Gabe waren neun von 30 Tieren verendet. Jedoch wiesen die überlebenden Tiere vier Tage nach der Gabe keine lokomotorischen Auffälligkeiten mehr auf (Tomas et al. 1999 b).

Bei WAG/Rij-Ratten mit implantierten Elektroden im fronto-parietalen Cortex wurden nach Schlundsondengabe von 0; 0,002; 0,008 oder 0,032 mol/kg KG der Trimethylbenzol-Isomere (ca. 0, 240, 960 oder 3846 mg/kg KG) oder Toluol geringere Effekte mit Trimethylbenzol beobachtet (Tomas et al. 1999 a).

#### 5.1.3 Dermale Aufnahme

Hierzu liegen keine Angaben mit reinen Trimethylbenzol-Isomeren vor. Daher werden Studien mit C9-Gemischen betrachtet, die Trimethylbenzol enthalten.

Mit **Shellsol A**, einem Lösungsmittel mit hauptsächlich C9-Aromaten und einem Siedepunkt ähnlich Testbenzin („white spirit“), wurde eine LD_50_ von > 3440 mg/kg KG (ca. > 4 ml/kg KG) ermittelt (Firth 2008).

Je drei weibliche und männliche Kaninchen erhielten einmalig 20 g **C9-Gemisch**/kg KG auf die radierte oder unbehandelte Haut aufgetragen. Nach 14 Tagen war ein männliches Tier verendet. Damit ist die LD_50_ > 20 g/kg (FMC Corp 1984).

#### 5.1.4 Intraperitoneale Aufnahme

Die LD_50_ nach intraperitonealer Gabe betrug bei männlichen und weiblichen BALB/c-Mäusen 4500 bzw. 3700 mg **1,3,5-Trimethylbenzol**/kg KG, 5000 bzw. 4100 mg **1,2,4-Trimethylbenzol**/kg KG, 3670 bzw. 2700 mg **1,2,3-Trimethylbenzol**/kg KG und 3750 bzw. 4065 mg Farbasol (96 % C9-Alkylbenzol, davon 46 % Trimethylbenzol, 40 % Ethyltoluol)/kg KG (Janik-Spiechowicz et al. 1998).

Je 20 männliche Wistar-Ratten mit implantierten Elektroden im fronto-parietalen Cortex und im Hippocampus erhielten eine intraperitoneale Gabe von **1,2,3-, 1,3,5- und 1,2,4-Trimethylbenzol** jeweils in Dosen von 0; 0,008 oder 0,016 mol/kg KG (0, 960 oder 1923 mg/kg KG). Bei Betrachtung der Korrelation zwischen EEG-Veränderungen und Blutkonzentrationen zeigte 1,2,3-Trimethylbenzol den stärksten Effekt (Tomas et al. 1999 c).

### 5.2 Subakute, subchronische und chronische Toxizität

#### 5.2.1 Inhalative Aufnahme

In subakuten und subchronischen Studien wurde gezeigt, dass hohe Expositionskonzentrationen ab 1000 ml Trimethylbenzol/m^3^ bei Ratten zu Augen- und Nasenreizungen, ZNS-Depression, erschwerter Atmung und reduzierter Körpergewichtszunahme führten (Greim 1998).

Die genauen Daten der nachfolgend beschriebenen Studien mit Trimethylbenzol sind in Tabelle 5 dargestellt.

Nach dreitägiger Ganzkörperexposition gegen 0, 125, 1250 oder 5000 mg **1,2,4-Trimethylbenzol**/m^3^ (ca. 0, 25, 250 oder 1000 ml/m^3^), acht Stunden pro Tag, traten bezüglich motorischer Aktivität und funktioneller Tests (FOB: „Functional Observational Battery“) ab 25 ml/m^3^ erste Effekte auf. Eine Steigerung der Griffstärke nach Exposition gegen 1000 ml/m^3^ erscheint den Autoren unplausibel (McKee et al. 2010).

Eine vierwöchige Ganzkörperexposition an fünf Tagen pro Woche und sechs Stunden täglich von je elf männlichen Wistar-Ratten gegen 0 oder 100 ml/m^3^**1,2,4-, 1,3,5- oder 1,2,3-Trimethylbenzol** führte bei allen drei Isomeren zu einer Abnahme der Latenzzeit im Test auf passives Vermeiden und einer Zunahme der Anzahl an Versuchen im Test auf aktives Vermeiden. Nur nach Exposition gegen 1,2,4- und 1,3,5-Trimethylbenzol kam es zu einer erhöhten lokomotorischen Aktivität (Open-Field-Test) und einer Zunahme der Latenzzeit bis zum Pfotenlecken nach thermischem Reiz (Hot-Plate-Test) (Gralewicz und Wiaderna 2001). Die Beobachtungsdauer der lokomotorischen Aktivität im Open-Field-Test betrug nur fünf Minuten. Dieser Zeitraum reicht nach Angabe weiterer Autoren nicht aus, um verlässliche Daten zu ermitteln, da eine Gewöhnung an Raum und Lichtverhältnisse nicht stattfinden konnte (Carola et al. 2002; US EPA 2016).

Je sechs bis acht männliche Wistar-Ratten wurden vier Wochen lang, an fünf Tagen pro Woche und sechs Stunden täglich, gegen **1,2,3- oder 1,2,4-Trimethylbenzol** in Konzentrationen von 0, 25, 100 oder 250 ml/m^3^ ganzkörperexponiert. 1,2,3-Trimethylbenzol bewirkte zwei Wochen nach Expositionsende bei 100 ml/m^3^ eine statistisch signifikant erhöhte lokomotorische Aktivität, jedoch nicht bei der höchsten Konzentration. Gegen 1,2,4-Trimethylbenzol exponierte Ratten zeigten keine statistisch signifikant erhöhte Aktivität (Lutz et al. 2010).

Eine vierwöchige Ganzkörperexposition an fünf Tagen pro Woche und sechs Stunden täglich von je 15 männlichen Wistar-Ratten gegen 0, 25, 100 oder 250 ml **1,2,4-Trimethylbenzol**/m^3^ bewirkte 35 bis 45 Tage nach der letzten Exposition bei 100 ml/m^3^ eine Latenzzeitabnahme beim passiven Vermeiden, wenn ein elektrischer Schock an der Pfote beim Heruntertreten erfolgte. Eine Latenzzeit-Zunahme bis zum Pfotenlecken nach thermischem Reiz (Hot-Plate-Test) und intermittierenden elektrischen Schocks an der Pfote wurde ab 100 ml/m^3^ beobachtet, jedoch ohne Konzentrationsabhängigkeit. Beim Verhalten im radialen Irrgarten und beim aktiven 2-Wege-Vermeidungsverhalten wurden keine statistisch signifikanten Effekte beobachtet (Gralewicz et al. 1997 b; Greim 1998).

Je zwölf männliche Wistar-Ratten wurden vier Wochen lang, an fünf Tagen pro Woche und sechs Stunden täglich, gegen 0, 25, 100 oder 250 ml **1,3,5-Trimethylbenzol**/m^3^ ganzkörperexponiert. Ab der niedrigsten Konzentration von 25 ml/m^3^ wurde im Test auf passives Vermeiden eine statistisch signifikant kürzere Latenzzeit nachgewiesen. Dieser Effekt verstärkte sich nicht bei höheren Konzentrationen. Im Test auf aktives Vermeiden zeigten die Tiere ab der niedrigsten Konzentration eine statistisch signifikant erhöhte Anzahl an Versuchen, die bei 250 ml/m^3^ noch etwas höher ausfiel und damit auf eine leichte Konzentrationsabhängigkeit hinweist. Die Latenzzeit der Reaktion auf den thermischen Reiz (Hot-Plate-Test), nach einem 24 Stunden zuvor erfolgten intermittierenden elektrischen Schock an der Pfote, war nur bei den Tieren, die gegen 100 ml/m^3^ exponiert waren, noch 50 Tage nach Expositionsende statistisch signifikant länger. Die Untersuchung des Verhaltens im radialen Irrgarten 14–18 Tage nach dem Expositionsende und der lokomotorischen Aktivität 25 Tage nach dem Expositionsende ergab keine statistisch signifikanten Effekte (Wiaderna et al. 2002). Der Test wird nicht zur MAK-Wert-Ableitung verwendet, sondern nur unterstützend zur Bewertung herangezogen.

Je 13–14 männliche Wistar-Ratten wurden vier Wochen lang, an fünf Tagen pro Woche und sechs Stunden täglich, gegen 0, 25, 100 oder 250 ml **1,2,3-Trimethylbenzol**/m^3^ ganzkörperexponiert. Der Test auf passive Vermeidung wurde 39–48 Tage nach dem Expositionsende durchgeführt und ergab bei 25 oder 100 ml/m^3^, nicht aber bei 250 ml/m^3^, eine statistisch signifikante Abnahme der Latenzzeit. Nur bei Exposition gegen 100 ml/m^3^ benötigten die Tiere häufigere Versuche in einem Testaufbau zum aktiven Vermeiden. Die Untersuchungen des Verhaltens im radialen Irrgarten 14–18 Tage nach dem Expositionsende und der lokomotorischen Aktivität 25 Tage nach dem Expositionsende ergaben keine statistisch signifikanten Effekte (Wiaderna et al. 1998). Beim Hot-Plate-Test wurde die Signifikanz der Pfotenlecken-Latenzzeit innerhalb einer Konzentrationsgruppe vor und nach einer zweiminütigen Serie elektrischer Schocks an der Pfote berechnet. Es fehlt hier die Signifikanz-Berechnung zwischen den einzelnen Konzentrationen. Der Test wird nicht zur Bewertung herangezogen.

Je 9–10 männliche Wistar-Ratten wurden vier Wochen lang, an fünf Tagen pro Woche und sechs Stunden täglich, gegen 0, 25, 100 oder 250 ml **1,2,4-Trimethylbenzol**/m^3^ ganzkörperexponiert. Einen Tag, 30 oder 120 Tage nach Expositionsende traten im EEG keine signifikanten Unterschiede auf. Eine bereits vor der Exposition implantierte Elektrode zeichnete nur in der höchsten Expositionsgruppe und nur 120 Tage nach Ende der Exposition eine statistisch signifikant reduzierte Anzahl an Spike-Wave-Entladungen auf (Gralewicz et al. 1997 a).

Eine vierwöchige Ganzkörperexposition von je fünf männlichen Wistar-Ratten an fünf Tagen pro Woche und sechs Stunden täglich gegen 0, 25, 97 oder 246 ml **1,2,3-Trimethylbenzol**/m^3^ (Reinheit > 90 %) verursachte keine veränderte Körpergewichtszunahme. Das relative Lebergewicht stieg um 4,4 % und das relative Nierengewicht um 8 % bei den Tieren mit der höchsten Exposition. Im Vergleich mit einer einmaligen sechsstündigen Exposition wurden im Blut und in Geweben statistisch signifikant niedrigere Konzentrationen beobachtet. Die Autoren führen das auf eine geringere Retention der Chemikalie in den Lungen der Ratten nach längerer Exposition zurück (siehe Abschnitt 5.1.1; Świercz et al. 2016).

Nach dreimonatiger Ganzkörperexposition an fünf Tagen in der Woche und sechs Stunden pro Tag gegen 0, 25, 100 oder 250 ml **1,2,4-Trimethylbenzol**/m^3^ wurden bei je zehn männlichen Ratten keine toxikologisch relevanten klinischen Befunde erhoben. Eine bronchoalveoläre Lavage wurde 24 Stunden nach Beendigung der Exposition bei je sechs Tieren durchgeführt. Die Gesamt-Protein-Konzentration, die Aktivitäten der Lactatdehydrogenase und der sauren Phosphatase sowie die Gesamtzahl der aus der Lunge herausgewaschenen Zellen in der bronchoalveolären Lavageflüssigkeit war in allen behandelten Gruppen statistisch signifikant erhöht. Die Zahl an Makrophagen nahm ab 100 ml/m^3^ statistisch signifikant zu. Eine Konzentrationsabhängigkeit zeigte sich in keinem Fall, sodass die Autoren eine Adaptation an die Exposition diskutierten (Greim 1998; Korsak et al. 1997). Es wurde nicht begründet, warum von den exponierten Tieren nur jeweils sechs Tiere untersucht wurden.

**Abb. 1:**
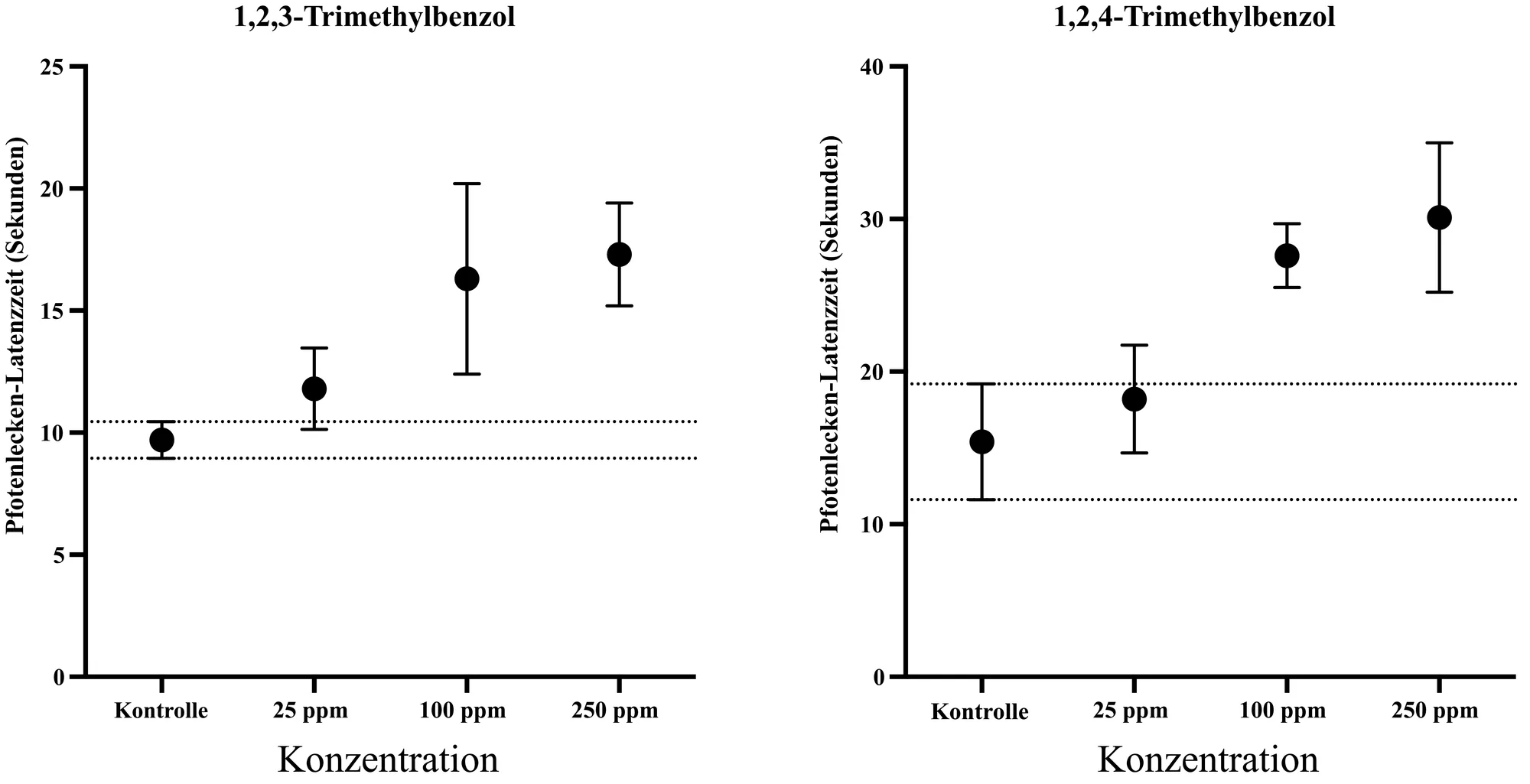
Latenzzeiten im Hot-Plate-Test bei Exposition gegen 1,2,3-Trimethylbenzol und 1,2,4-Trimethylbenzol von Korsak und Rydzyński (1996). Mittelwerte erstellt mit nachträglich aus der Standardabweichung berechneten 95-%-Konfidenzgrenzen bei angenommener Normalverteilung der Daten (van Thriel 2024)

Je neun oder zehn männliche Wistar-Ratten wurden drei Monate lang, an fünf Tagen pro Woche und sechs Stunden täglich, gegen 0, 25, 100 oder 250 ml **1,2,4-Trimethylbenzol**/m^3^ ganzkörperexponiert. Eine Leistungsminderung um 10 % wurde im Rotarod-Test auf Bewegungskoordination nach vier Wochen Exposition gegen 25 ml/m^3^ beobachtet, jedoch ohne Zunahme des Effekts nach acht oder 13 Wochen. Der Effekt war nicht statistisch signifikant im Fisher-Exact-Test. Ab 100 ml/m^3^ waren die Latenzzeiten im Hot-Plate-Test (Pfotenlecken) verlängert (Abbildung 1). Die Leistungsminderung im Rotarod-Test bei 250 ml/m^3^ blieb auch in der 14-tägigen Nachbeobachtungszeit bestehen, war aber nicht statistisch signifikant (Korsak und Rydzyński 1996). Beim Rotarod-Test wurde keine Standardabweichung angegeben. Eine genaue Beschreibung zur Häufigkeit der Testung jedes einzelnen Tieres fehlt. Die NOAEC beträgt 25 ml/m^3^.

Drei Monate lang, an fünf Tagen pro Woche und sechs Stunden täglich, wurden männliche Wistar-Ratten gegen 0 (n = 30), 25 (n = 20), 100 (n = 10) oder 250 (n = 10) ml **1,2,3-Trimethylbenzol**/m^3^ ganzkörperexponiert. Die Latenzzeiten im Hot-Plate-Test (Pfotenlecken) waren nur bei den Expositionskonzentrationen 25 und 250 ml/m^3^ statistisch signifikant (Kruskal-Wallis-Test) verlängert. Eine Leistungsminderung im Rotarod-Test auf Bewegungskoordination um 20 % wurde nach acht Wochen ab 25 ml/m^3^ beobachtet, jedoch ohne Zunahme nach 13 Wochen. Der Effekt war nicht statistisch signifikant im Fisher-Exact-Test. Auch nach einer 14-tägigen Nachbeobachtungszeit der 250-ml/m^3^-Gruppe (zehn Tiere) blieb die Leistungsminderung im Rotarod-Test bestehen (Korsak und Rydzyński 1996). Eine nachträglich von US EPA (2016) mit einem Ad-hoc-t-Test durchgeführte Signifikanzberechnung der verlängerten Latenzzeit im Hot-Plate-Test nach Exposition gegen 100 ml/m^3^ ergab p < 0,01. Die Varianz der Ergebnisse hängt von der Anzahl der Tiere ab, sodass die unterschiedlichen Tierzahlen hier die Standardabweichungen und Konfidenzgrenzen bei den beiden untersuchten Isomeren beeinflussen (Abbildung 1). Beim Rotarod-Test wurde keine Standardabweichung angegeben. Eine genaue Beschreibung zur Häufigkeit der Testung jedes einzelnen Tieres fehlt.

Nach Angaben der Autoren wurde der Hot-Plate-Test (Korsak und Rydzyński 1996) unmittelbar nach Beendigung der Exposition durchgeführt. Auffallend war jedoch, dass die beiden Kontrollgruppen Unterschiede in der Latenzzeit um ca. 50 % aufwiesen. Die Autoren wurden diesbezüglich um Auskunft gebeten und erklärten dies durch die verschiedenen Generationen F2 bzw. F7 mit denen die Tests durchgeführt wurden (Greim 1998). Es fehlen entsprechende Angaben, ob die Durchführung des Rotarod-Tests unmittelbar nach dem Hot-Plate-Test mit derselben Tierkohorte stattfand. Dies könnte ein potenzieller Störfaktor sein (US EPA 2016).

Je zehn weibliche und männliche Wistar-Ratten wurden drei Monate lang, an fünf Tagen pro Woche und sechs Stunden täglich, gegen 0, 129 ± 18 mg, 492 ± 62 oder 1207 ± 76 mg **1,2,4-Trimethylbenzol**/m^3^ (ca. 0, 26, 99 oder 240 ml 1,2,4-Trimethylbenzol/m^3^) ganzkörperexponiert. Die Effektschwere in der Lunge wurde mit dem Kruskal-Wallis-Test ausgewertet. Je zehn Tiere der höchsten Konzentrationsgruppe wurden 14 Tage nachbeobachtet. Bei einer Konzentration von 99 ml/m^3^ wurden histologische Effekte in der Lunge der männlichen Tiere beobachtet, die jedoch bei der hohen Konzentration nicht mehr auftraten. Bei den Tieren der höchsten Konzentrationsgruppe wurden veränderte Blutwerte nachgewiesen. Die Bestimmung der Organgewichte, der Parameter der klinischen Chemie 18 Stunden nach der letzten Exposition (außer einer Erhöhung der Sorbitdehydrogenase-Aktivität der männlichen Tiere) und weitere histologische Untersuchungen waren ohne substanzbedingten Befund. Die mikroskopische Untersuchung des oberen Atemtrakts (Mukosa von Nase und Luftröhre) ergab ebenfalls keine substanzbedingten Befunde (Korsak et al. 2000 b). Die NOAEC beträgt 26 ml/m^3^. Neurotoxizität wurde nicht getestet. Die Einzeltierdaten liegen nicht vor.

In einer weiteren Studie dieser Arbeitsgruppe wurden je zehn weibliche oder männliche Imp:Wistar-Ratten drei Monate lang, an fünf Tagen pro Woche und sechs Stunden täglich, gegen 0, 128 ± 10 mg, 532 ± 38 mg oder 1269 ± 36 mg **1,2,3-Trimethylbenzol**/m^3^ (ca. 0, 26, 110 oder 260 ml 1,2,3-Trimethylbenzol/m^3^) ganzkörperexponiert. Ab der niedrigsten Konzentration von 26 ml/m^3^ traten Effekte in der Lunge auf, die nur bei dieser Konzentration beobachtet wurden oder sich nicht konzentrationsabhängig verstärkten. Ab 110 ml/m^3^ war die Anzahl von Becherzellen in der Lunge der Tiere statistisch signifikant erhöht. In der Lunge der männlichen Tiere der 260-ml/m^3^-Gruppe wurden interstitielle Infiltrationen beobachtet. Die mikroskopische Untersuchung des oberen Atemtrakts (Mukosa von Nase und Luftröhre) ergab keine substanzbedingten Befunde (Korsak et al. 2000 a). Neurotoxizität wurde nicht getestet. Einzeltierdaten liegen nicht vor. Effekte in der Lunge, die nur in der niedrigsten Expositionsgruppe beobachtet wurden oder sich nicht konzentrationsabhängig verstärkten, werden nicht als advers angesehen.

##### Gemische

Nach dreitägiger Exposition an acht Stunden pro Tag von je acht männlichen Wistar-Ratten gegen 0, 600, 2400 oder 4800 mg **Testbenzin** („white spirit“, C9–C12 mit 21,3 % aromatischen Alkylverbindungen)/m^3^ wurden bei den Tieren der mittleren und hohen Expositionsgruppe statistisch signifikant längere Latenzzeiten im Visual-Discrimination-Performance-Test (Lernleistung) beobachtet. Es traten keine klinischen Auffälligkeiten auf. Das Körpergewicht war ab 2400 mg/m^3^ statistisch signifikant erniedrigt. Die mittleren Trimethylbenzol-Konzentrationen im Blut betrugen < 20 ng/ml (Nachweisgrenze), 158, 810 bzw. 3870 ng/ml und im Gehirn < 150 ng/g (Nachweisgrenze), 420, 2212 bzw. 8788 ng/g (Lammers et al. 2007).

Je 20 männliche Sprague-Dawley-Ratten wurden fünf, neun oder 13 Wochen lang, an fünf Tagen pro Woche und sechs Stunden täglich, gegen 0, 101, 432 oder 1320 ml **high flash aromatic Naphtha (HFAN)**/m^3^ ganzkörperexponiert. HFAN enthielt 55 % Trimethylbenzol, 28 % Ethyltoluol, 3,2 % Xylol, 2,74 % Isopropylbenzol, 3,97 % *n*-Propylbenzol und 6,19 % Verbindungen mit 10 oder mehr C-Atomen. Bis zur höchsten Konzentration traten keine Unterschiede zur Kontrollgruppe bezüglich motorischer Aktivität und funktioneller Tests (FOB) auf. Gemessen wurde die Griffstärke der Vorder- und Hinterextremitäten, Antwort auf ein Geräuschsignal, thermische Reize im Hot-Plate-Test und Spreizfähigkeit der Pfoten (siehe auch Tabelle 5; Douglas et al. 1993; Api 1988 in Greim 1998).

Nach Exposition (78–150 Tage) gegen ein **C9/C10-Gemisch** (Destillat aus Naphtha bei 155–200 °C) in Konzentrationen von 0, 50, 200, 616 oder 1000 ml/m^3^, 18 Stunden pro Tag und sieben Tage die Woche, entwickelten Ratten ab 616 ml/m^3^ Stauungen in der Leber und Lunge, vergrößerte Milz, Blutungen in den Nieren und reduzierte Körpergewichtszunahmen (Nau et al. 1966).

**C9-Aromatengemische** mit 44,8–55 % Gesamt-Trimethylbenzol verursachten in Inhalationsstudien (13 Wochen bis zwölf Monate) bis zu einer Konzentration von 194 ml/m^3^ (ca. 87 ml Trimethylbenzol/m^3^) bei Ratten keine Effekte. Eine Exposition gegen 366 ml C9-Aromatengemisch/m^3^ (164 ml Trimethylbenzol/m^3^) führte bei den männlichen Tieren nach sechs und zwölf Monaten zu einer verstärkten Makrophageninfiltration in der Lunge und Verdickung der Alveolenwände. Die weiteren bei höheren Konzentrationen aufgetretenen Effekte sind in Greim (1998) ausführlich dargestellt.

Je drei Rhesusaffen wurde sieben Stunden täglich, fünf Tage pro Woche über einen Zeitraum von 18 Wochen gegen 0, 50 oder 200 ml **C9/C10-Gemisch** (Destillat aus Naphtha bei 155–200 °C)/m^3^ exponiert. Bei den Tieren der 50-ml/m^3^-Gruppe wurden erhöhte Hämatokritwerte und eine Umkehrung des Verhältnisses zwischen polymorphkernigen Neutrophilen und Lymphozyten (k. w. A) beobachtet. Die höher exponierten Tiere entwickelten in der ersten Expositionswoche einen Tremor, der im Verlauf der weiteren Exposition abnahm. Am Ende der Expositionszeit wirkten die Tiere erschöpft und sediert. Die weißen Blutzellen und die myelozytische und erythrozytische Aktivität des Knochenmarks nahm ab (Nau et al. 1966).

**Tab. 5:** Toxizität von Trimethylbenzol bei Ratten nach wiederholter inhalativer Exposition

**Spezies,** **Stamm,** **Anzahl pro Gruppe**	**Exposition**	**Befunde^a)^**	**Literatur**
Ratte, Wistar, 8 ♂	3 d, 0, 125, 1250, 5000 mg **1,2,4-Trimethylbenzol**/m^3^ (ca. 0, 25, 250 oder 1000 ml/m^3^), 8 h/d, 3 d/Wo, Reinheit > 98 %, Ganzkörper	**bei 25 ml/m^3^**: Griffstärke der Vorderextremitäten ↓; **ab 250 ml/m^3^**: Anzahl Bewegungen ↓ (n. sign.), repetitive „inter-trial responses“ ↓ (Visual-Discrimination-Performance-Test); **1000 ml/m^3^**: KG ↓ (um 5 %), Griffstärke der Vorderextremitäten ↑, zurückgelegte Gesamtstrecke ↓, Anzahl Bewegungen ↓	McKee et al. 2010
Ratte, Wistar, 11 ♂, Kontrolle 10 ♂	4 Wo, 0, 100 (± 10) ml **1,3,5-Trimethylbenzol**/m^3^ oder 0, 100 (± 10) ml **1,2,4-Trimethylbenzol**/m^3^, 6 h/d, 5 d/Wo, Reinheit k. A., Ganzkörper	**100 ml/m^3^**: lokomotorische Aktivität ↑ (Open-Field-Test), passives Vermeiden Latenzzeit ↓, Hot-Plate-Test 54,5 °C: Pfotenlecken Latenzzeit ↑, aktives Vermeiden ↑	Gralewicz und Wiaderna 2001
Ratte, Wistar, 11 ♂, Kontrolle 10 ♂	4 Wo, 0, 100 (± 10) ml **1,2,3-Trimethylbenzol**/m^3^, 6 h/d, 5 d/Wo, Reinheit k. A., Ganzkörper	**100 ml/m^3^**: passives Vermeiden Latenzzeit ↓, aktives Vermeiden ↑	Gralewicz und Wiaderna 2001
Ratte, Wistar, 15 ♂	4 Wo, 0, 25, 100, 250 ml **1,2,4-Trimethylbenzol**/m^3^, 6 h/d, 5 d/Wo, Reinheit k. A., Ganzkörper	**bei 100 ml/m^3^**: Pflegeverhalten ↑ (Open-Field-Test); **ab 100 ml/m^3^**: passives Vermeiden 35–45 Tage nach Expositionsende Latenzzeit ↓ (nicht konzentrationsabhängig), Hot-Plate-Test 54,5 °C: Pfotenlecken Latenzzeit ↑ (nicht konzentrationsabhängig)	Gralewicz et al. 1997 b; Greim 1998
Ratte, Wistar, 6–8 ♂	4 Wo, 0, 25, 100, 250 ml **1,2,3-Trimethylbenzol**/m^3^, 6 h/d, 5 d/Wo, Reinheit 97 %, Ganzkörper	**bei 100 ml/m^3^**: lokomotorische Aktivität ↑	Lutz et al. 2010
Ratte, Wistar, 6–8 ♂	4 Wo, 0, 25, 100, 250 ml **1,2,4-Trimethylbenzol**/m^3^, 6 h/d, 5 d/Wo, Reinheit 97 %, Ganzkörper	**250 ml/m^3^**: **NOAEC**	Lutz et al. 2010
Ratte, Wistar, 12 ♂	4 Wo, 0, 25, 100, 250 ml **1,3,5-Trimethylbenzol**/m^3^, 6 h/d, 5 d/Wo, Reinheit k. A., Ganzkörper	**ab 25 ml/m^3^**: passives Vermeiden Latenzzeit ↓ (nicht konzentrationsabhängig), aktives Vermeiden ↑ (gering konzentrationsabhängig); **bei 100 ml/m^3^**: Hot-Plate-Test 54,5 °C: Pfotenlecken Latenzzeit ↑	Wiaderna et al. 2002
Ratte, Wistar, 13–14 ♂	4 Wo, 0, 25, 100, 250 ml **1,2,3-Trimethylbenzol**/m^3^, 6 h/d, 5 d/Wo, Reinheit k. A., Ganzkörper	**ab 25 ml/m^3^**: passives Vermeiden Latenzzeit ↓ (nicht konzentrationsabhängig); **bei 100 ml/m^3^**: aktives Vermeiden ↑, Hot-Plate-Test 54,5 °C: Pfotenlecken Latenzzeit ↑	Wiaderna et al. 1998
Ratte, Wistar, 5 ♂	4 Wo, 0, 25, 97, 246 ml **1,2,3-Trimethylbenzol**/m^3^, 6 h/d, 5 d/Wo, Reinheit > 90 %, Ganzkörper	**246 ml/m^3^**: rel. Lebergewicht ↑ (um 4,4 %), rel. Nierengewicht ↑ (um 8 %)	Świercz et al. 2016
Ratte, Wistar, 4 ♂	4 Wo, 0, 25, 100, 250 ml **1,2,4-Trimethylbenzol**/m^3^, 6 h/d, 5 d/Wo, Reinheit > 97 %, Ganzkörper	**ab 25 ml/m^3^**: Anreicherung: Gehirn ↑ (0,45 ± 0,05 mg/kg), Leber ↑ (0,45 ± 0,15 mg/kg), Lunge ↑ (0,47 ± 0,2 mg/kg); **ab 100 ml/m^3^**: Anreicherung: Gehirn ↑ (2,82 mg/kg), Leber ↑ (3,00 mg/kg), Lunge ↑ (3,74 mg/kg); **250 ml/m^3^**: Anreicherung: Gehirn ↑ (18,63 mg/kg), Leber ↑ (22,47 mg/kg), Lunge ↑ (22,47 mg/kg (sic!))	Świercz et al. 2003, siehe auch Abschnitt 3.1
Ratte, Wistar, 6 ♂	3 Mo, 0, 25, 100, 250 ml **1,2,4-Trimethylbenzol**/m^3^, 6 h/d, 5 d/Wo, Reinheit > 97 %, Ganzkörper	**ab 25 ml/m^3^**: BALF: Gesamtzellzahl ↑, Gesamtprotein ↑, LDH ↑, saure Phosphatase ↑ (alle Effekte nicht konzentrationsabhängig), Mucoprotein ↓; **ab 100 ml/m^3^**: BALF: Makrophagen ↑ (nicht konzentrationsabhängig)	Greim 1998; Korsak et al. 1997
Ratte, Wistar, 9 oder 10 ♂	3 Mo, 0, 25, 100, 250 ml **1,2,4-Trimethylbenzol**/m^3^, 6 h/d, 5 d/Wo, 14 d Nachbeobachtung der Expositionsgruppe 250 ml/m^3^, Reinheit > 97 %, Ganzkörper	**25 ml/m^3^**: **NOAEC,** Rotarod-Test: Koordination ↓ (10 % ab 4. Wo, n. sign. im Fisher-Exact-Test); **ab 100 ml/m^3^**: Hot-Plate-Test 54,5 °C: Pfotenlecken Latenzzeit ↑, Rotarod-Test: Koordination ↓ (20 % ab 8. Wo, n. sign. im Fisher-Exact-Test); **250 ml/m^3^**: Rotarod-Test: Koordination ↓ (40 % ab 8. Wo, statistisch signifikant im Fisher-Exact-Test); **14 d Nachbeobachtung: ** **250 ml/m^3^**: Rotarod-Test: Koordination ↓, n. sign.	Greim 1998; Korsak und Rydzyński 1996
Ratte, Wistar, 10 ♂, Hot-Plate-Test: 30 ♂ (Kontrolle), 20 ♂ (25 ml/m^3^)	3 Mo, 0, 25, 100, 250 ml **1,2,3-Trimethylbenzol**/m^3^, 6 h/d, 5 d/Wo, 14 d Nachbeobachtung der Expositionsgruppe 250 ml/m^3^, Reinheit 90–95 %, Ganzkörper	**25 ml/m^3^**: Hot-Plate-Test 54,5 °C: Pfotenlecken Latenzzeit ↑, Rotarod-Test: Koordination ↓ (20 % ab 8. Wo, n. sign. im Fisher-Exact-Test); **ab 100 ml/m^3^**: Rotarod-Test: Koordination ↓ (40 % ab 13. Wo, statistisch signifikant im Fisher-Exact-Test); **250 ml/m^3^**: Hot-Plate-Test 54,5 °C: Pfotenlecken Latenzzeit ↑; **14 d Nachbeobachtung: ** **250 ml/m^3^**: Rotarod-Test: Koordination ↓	Greim 1998; Korsak und Rydzyński 1996; US EPA 2016
Ratte, Wistar, 10 ♂, 10 ♀	3 Mo, 0, 26, 99, 240 ml **1,2,4-Trimethylbenzol**/m^3^, 6 h/d, 5 d/Wo, 14 d Nachbeobachtung der Expositionsgruppe 240 ml/m^3^, Reinheit > 97 %, Ganzkörper	**26 ml/m^3^**: **NOAEC;** **ab 26 ml/m^3^**: ♂: Sorbitdehydrogenase ↑ (nicht konzentrationsabhängig); **ab 99 ml/m^3^**: ♂/♀: Lunge: Interstitielle Infiltrationen ↑ mäßig bis deutlich (♂: nur diese Konzentration), ♂: Lunge: Proliferation des peribronchialen Gewebes (nur diese Konzentration), alveoläre Makrophagen ↑ mäßig, ♀: Blutgerinnung ↓ (nicht konzentrationsabhängig); **240 ml/m^3^**: ♂/♀: Leukozyten ↑, ♂: Erythrozyten ↓, ♀: Retikulozyten ↓, Lunge: alveoläre Makrophagen ↑; **14 d Nachbeobachtung:** **240 ml/m^3^**: ♂: Erythrozyten ↓	Korsak et al. 2000 b
Ratte, Imp:Wistar, 10 ♂, 10 ♀	3 Mo, 0, 26, 110, 260 ml **1,2,3-Trimethylbenzol**/m^3^, 6 h/d, 5 d/Wo, 14 d Nachbeobachtung der Expositionsgruppe 260 ml/m^3^, Reinheit > 97 %, Ganzkörper	**26 ml/m^3^**: **NOAEC;** **ab 26 ml/m^3^**: ♀: Retikulozyten ↑ (nicht konzentrationsabhängig); **ab 110 ml/m^3^**: ♀: Lunge: Becherzellen ↑ mäßig, rel. Milzgewicht ↓, Neutrophile ↓, AP ↑; **260 ml/m^3^**: ♂/♀: Lymphozyten ↑, Neutrophile ↓, ♂: rel. Lebergewicht ↑ (um 9 %), Erythrozyten ↓, Retikulozyten ↑, Sorbitdehydrogenase ↑, Lunge: interstitielle Infiltrationen ↑ deutlich, ♀: Lunge: Becherzellen ↑ deutlich, ALT ↓; **14 d Nachbeobachtung:** **260 ml/m^3^**: ♂/♀: Retikulozyten ↑	Korsak et al. 2000 a
Ratte, Sprague Dawley, 20 ♂	3 Mo, 0, 101, 432, 1320 ml **HFAN**/m^3^ (ca. 0, 56, 249, 726 ml Trimethylbenzol/m^3^)	**ca. 249 ml Trimethylbenzol/m^3^**: **NOAEC;** **ca. 726 ml Trimethylbenzol/m^3^**: KG ↓, NOAEC für motorische Aktivität, Hot-Plate-Test und funktionelle Tests (FOB)	Douglas et al. 1993 (API 1988c in Greim 1998)

a) wenn nicht anders angegeben, sind die aufgeführten Veränderungen statistisch signifikant

ALT: Alaninaminotransferase; AP: alkalische Phosphatase; BALF: bronchoalveoläre Lavageflüssigkeit; d: Tag; FOB: funktionelle Tests („functional observation battery“); h: Stunde; HFAN: high flash aromatic Naphtha (Ethyltoluol 28 % und Trimethylbenzol 55 %); LDH: Laktatdehydrogenase; Mo: Monat; n. sign.: nicht statistisch signifikant; rel.: relativ; Wo: Woche

##### Fazit:

Der empfindlichste Endpunkt der Wirkung von Trimethylbenzol ist die Neurotoxizität. Statistisch signifikante geringe Effekte treten ab 25 ml/m^3^ im Hot-Plate-Test (analgetische Wirkung) und ab 100 ml/m^3^ im Koordinationstest (Rotarod-Test) bei 1,2,3-Trimethylbenzol auf. Statistisch signifikante Wirkungen im Hot-Plate-Test und im Rotarod-Test treten ab 100 ml/m^3^ bzw. bei 250 ml/m^3^ mit 1,2,4-Trimethylbenzol auf (Korsak und Rydzyński 1996). Die nach vierwöchiger Exposition gegen 25 ml/m^3^ 1,2,3-Trimethylbenzol und 1,3,5-Trimethylbenzol beginnenden geringen Veränderungen an passivem und aktivem Vermeiden waren nicht konzentrationsabhängig. Es bleibt unklar, ob die Testung im Rotarod-Test die Ergebnisse des Hot-Plate-Tests beeinflusst hat. Zudem wurden die Ergebnisse nicht von einer anderen Arbeitsgruppe repliziert und die Adversität der analgetischen Wirkung bei 25 ml/m^3^ ist unklar. Deshalb wird insgesamt 25 ml/m^3^ als NOAEC angesehen.

#### 5.2.2 Orale Aufnahme

Nach einer 14-tägigen Schlundsondengabe von 0, 60, 150 oder 600 mg **1,3,5-Trimethylbenzol**/kg KG und Tag (Reinheit 99,2 %) an je zehn Sprague-Dawley-Ratten wurden reversible Anstiege von Cholesterin und relativem Lebergewicht in den beiden höchsten Dosisgruppen nachgewiesen mit 9 % bzw. 25,6 % bei weiblichen Tieren und 6 % bzw. 22 % bei männlichen Tieren. Geringe, nach 14 Tagen reversible, zentrilobuläre Hypertrophie von Hepatozyten zeigten alle männlichen Tiere und drei von zehn weiblichen Tieren der höchsten Dosisgruppe. Diese Effekte werden auf die Induktion mikrosomaler Enzyme zurückgeführt. Ein reversibler Anstieg der weißen Blutkörperchen mit entsprechender Zunahme der Neutrophilen und Lymphozyten wurde bei männlichen Ratten bei 600 mg/kg KG und Tag beobachtet. Die Tiere dieser Dosisgruppe zeigten zudem gerötete Nasen, Fellverfärbung, Alopezie, feuchtes Fell und erhöhten Speichelfluss. Bei den männlichen Kontrolltieren wiesen 30 % und bei den weiblichen 60 % eine chronische Leberentzündung auf (Adenuga et al. 2014; IITRI 1995 a).

Sprague-Dawley-Ratten wurden nach 28-tägigen Schlundsondengaben von **1,2,3-Trimethylbenzol** oder **1,2,4-Tri­methylbenzol** in Dosen von 0, 30, 100, 300 oder 1000 mg/kg KG und Tag untersucht. Bei Gabe von 1,2,3-Trimethylbenzol wurden eine Zunahme des Leber- und Nierengewichts zusammen mit einer Abnahme des Thymusgewichts, klinisch-chemischen Veränderungen und, nur bei der höchsten Dosis, hyaline Tröpfchen und eosinophile Veränderungen in den Nieren männlicher Tiere beobachtet. Mit 1,2,4-Trimethylbenzol wurden ebenfalls Zunahmen des Leber- und Nierengewichts, jedoch keine hämatologischen Veränderungen oder Anomalien bei der Sektion beobachtet. Laut der Autoren lag der NOAEL bei 100 mg/kg KG und Tag (k. w. A.; Firth 2008). Die Originalstudie liegt nicht vor.

Nach 28-tägiger Schlundsondengabe von 0, 100, 300 oder 1000 mg **1,2,3-Trimethylbenzol**/kg KG und Tag an Ratten traten bereits ab der niedrigsten Dosis Veränderungen der klinisch-chemischen Parameter, reduziertes Thymusgewicht, erhöhtes Organgewicht von Leber und Nieren sowie hepatozelluläre Schwellungen auf. Eine Nachfolgestudie mit 0, 3, 10 oder 30 mg/kg KG und Tag ergab keine Effekte (k. w. A.; Firth 2008). Die Originalstudie liegt nicht vor.

In einer Studie nach OECD-Prüfrichtlinie 408 mit Schlundsondengaben von 0, 50, 200 oder 600 mg **1,3,5-Trimethylbenzol**/kg KG und Tag in Maiskeimöl an je zehn männliche und weibliche Sprague-Dawley-Ratten an fünf Tagen pro Woche über einen Zeitraum von 90 Tagen und einer Nachbeobachtungszeit von 28 Tagen traten nur bei den Tieren der beiden höchsten Dosisgruppen Effekte auf. So wurden neben erhöhten Serumphosphat- und Cholesterinwerten ein um 16 % bzw. 26 % erhöhtes relatives Lebergewicht und bei den männlichen Tieren zudem ein erhöhtes relatives Nierengewicht jeweils ohne histopathologische Veränderungen beobachtet. Die bei der höchsten Dosis berichteten Wirkungen wurden von den Autoren auf eine adaptive Reaktion (Enzyminduktion) der Prüfsubstanz zurückgeführt. Die Körpergewichtszunahme bei den männlichen Tieren der hohen Dosierung war um 11 % reduziert. Die Tiere mit hoher Dosierung zeigten zudem Fellverfärbung, feuchtes Fell und erhöhten Speichelfluss. Alle beobachteten Effekte waren reversibel. Eine mikroskopische Untersuchung erfolgte bei den Tieren der niedrigen und mittleren Dosis nur beim Lungengewebe. Viele Kontrolltiere zeigten Lebereffekte wie chronische Entzündung (sechs weibliche und vier männliche Tiere) und Niereneffekte wie Mineralisierung (sieben weibliche Tiere) und Nephropathie (drei männliche Tiere). Bei den Tieren der hohen Dosis traten die Lebereffekte in ähnlicher Größenordnung auf (k. w. A.; Adenuga et al. 2014; IITRI 1995 b). Das Leberwachstum ist damit auf die Induktion mikrosomaler Enzyme und das erhöhte Nierengewicht bei männlichen Ratten wahrscheinlich auf eine α2u-Globulin-bedingte Nephropathie zurückzuführen.

#### 5.2.3 Dermale Aufnahme

Die Auftragung von 0,1–0,5 g **C9/C10-Gemisch** (Destillat aus Naphtha bei 155–200 °C), dreimal pro Woche, 50 Wochen lang auf die Haut von Mäusen (insgesamt 10,62 g pro Maus) bewirkte fokale Blutungen (12 %), Pigmentierungen (10 %), Entzündungen (16 %) und Atelektase (7 %) in der Lunge. In der Leber wurden fokale nekrotische Stellen (7 %) sowie Amyloidose (3,5 %), dies auch in der Milz (57 %), beobachtet. In der Niere traten kortikale Vernarbungen (35,5 %), Sklerose (27 %) und Nekrosen in den renalen Papillen (12 %) auf. Die Anzahl der weißen Blutzellen war am Ende der Exposition verdoppelt und es wurde ein Anstieg an polymorphkernigen Leukozyten gefunden (Nau et al. 1966). Die Effekte an der Haut werden in Abschnitt 5.3.1 beschrieben.

### 5.3 Wirkung auf Haut und Schleimhäute

#### 5.3.1 Haut

Die Auftragung von 0,1–0,15 g **C9/C10-Gemisch** (Destillat aus Naphtha bei 155–200 °C), dreimal pro Woche, 50 Wochen lang auf die Haut von Mäusen (insgesamt 10,62 g pro Maus) bewirkte Hyperkeratose (31 %), Atrophie der Epidermis (24 %), Entzündungsreaktionen (41 %), Parakeratose (23 %) und Ulzerationen (25 %) (Nau et al. 1966).

Eine dermale Applikation von unverdünntem **1,2,4- **oder** 1,3,5-Trimethylbenzol** auf die rasierte Rückenhaut von Kaninchen führte zu Erythemen (Greim 1998).

Ein primärer Reizindex von 2,25 (maximaler primärer Reizindex = 8) wurde 24 und 76 Stunden nach okklusiver Auftragung von 0,5 ml eines **C9-Gemisches** mit den drei Trimethylbenzol-Isomeren auf die abradierte oder unbehandelte Haut von je drei männlichen und weiblichen Kaninchen erhalten (FMC Corp 1984).

Bei okklusiver Auftragung von 0,5 ml **1,2,4-Trimethylbenzol** (Reinheit 98 %) auf die geschorene, abradierte oder unbehandelte Haut von sechs weißen Neuseeländer-Kaninchen betrug der Reizindex nach 24 und 76 Stunden 3,7. Die Substanz wird von den Autoren als nicht reizend eingeschätzt, da der Wert unter 5 liegt (MB Research Laboratories 1982 b).

In einer Studie zur Hautirritation wurde die Haut von fünf bis sechs weißen Neuseeländer-Kaninchen für vier Stunden mit 0,5 ml unverdünntem **1,3,5-Trimethylbenzol** oder einer 50%igen, 25%igen, 10%igen oder 5%igen (G/G)-Lösung in Mandelöl okklusiv exponiert. 24, 48 und 72 Stunden nach Ende der Exposition wurde die Haut auf Erytheme und Ödeme untersucht. **1,3,5-Trimethylbenzol**-Konzentrationen von mehr als 50 % wirkten irritierend auf die Haut. Die NOAEC, definiert als die Konzentration, bei der der mittlere Reizwert für Erytheme unter 2 liegt, betrug 25 % (Jacobs et al. 1987).

Je drei Rhesusaffen wurden sieben Stunden täglich, fünf Tage pro Woche, über einen Zeitraum von 26 Tagen gegen 0, 50 oder 200 ml **C9/C10-Gemisch** (Destillat aus Naphtha bei 155–200 °C)/m^3^ inhalativ ganzkörperexponiert. Die Tiere wiesen Hautreizungen mit Haarverlusten auf, sowie die Bildung von trockenen und ledrig wirkenden Hautarealen (siehe Abschnitt 5.2.1, Nau et al. 1966). Es ist unklar, ob eine Kontrollgruppe mitgeführt wurde. Möglicherweise dienten die Tiere vor der Behandlung als Kontrolle.

**1,2,4- **und** 1,3,5-Trimethylbenzol** sind nach dem global harmonisierten System in die Kategorie 2 für hautreizende Stoffe eingestuft. Es existiert kein Registrierungsdossier für 1,2,3-Trimethylbenzol (ECHA 2023, 2024).

#### 5.3.2 Auge

In einer Studie zur Augenreizung traten bei sechs Kaninchen, denen 0,1 ml **1,2,4-Trimethylbenzol** appliziert wurde, eine leichte Rötung der Konjunktiva und Chemosis auf, während 1,3,5-Trimethylbenzol als nicht reizend bewertet wurde (Greim 1998).

Neun Kaninchen wurde einmalig 0,1 ml eines unverdünnten **C9-Gemisches** ins Auge gegeben. Innerhalb von 24 Stunden wurde bei drei Kaninchen viermaliges Spülen des Auges durchgeführt. Die mittleren Reizwerte betrugen nach einem Tag 2,3 bzw. 2,7, nach vier Tagen 3,0 bzw. 1,3 und nach sieben Tagen 0,7 bzw. 0,0 (Score 1–4) (FMC Corp 1984).

Bei sechs weißen Neuseeländer-Kaninchen führte die Applikation von 0,1 ml **1,2,4-Trimethylbenzol** in den Konjunktivalsack des Auges nur am ersten Tag zu einer Iritis bei einem Tier und bei einem weiteren Tier zu Konjunktivitis ab dem dritten Tag nach der Behandlung. Die Autoren bewerten den Stoff als nicht reizend am Auge (MB Research Laboratories 1982 b).

Bei sechs Kaninchen führte die Applikation von 0,1 ml **1,2,4-Trimethylbenzol** in ein Auge zu einer leichten Reizung. Die Bewertung nach dem Draize-Index (Maximum 110) ergab 6 nach 24 Stunden, 4 nach 48 Stunden und 2 nach 72 Stunden. Danach waren die Effekte reversibel (Dupont 1983).

Nach 15-maliger sechsstündiger Ganzkörper-Exposition von Ratten gegen 1000 ml **1,2,4-Trimethylbenzol**/m^3^ traten leichte Augen- und Nasenirritationen auf (Greim 1998).

**1,2,4- und 1,3,5-Trimethylbenzol** sind nach dem global harmonisierten System in die Kategorie 2 für augenreizende Stoffe eingestuft. Es existiert kein Registrierungsdossier für 1,2,3-Trimethylbenzol (ECHA 2023, 2024).

### 5.4 Allergene Wirkung

#### 5.4.1 Hautsensibilisierende Wirkung

##### 5.4.1.1 Tierexperimentelle Untersuchungen

Es liegt nur ein sekundär und unvollständig berichteter Maximierungstest an Meerschweinchen mit unverdünntem **1,2,4-Trimethylbenzol** vor, der negativ verlief (ECHA 2023), aber aufgrund fehlender weiterer Informationen nicht zur Bewertung herangezogen werden kann.

Es liegen zwei weitere Maximierungstests mit Produktgemischen vor, die Trimethylbenzole enthalten. Ein Maximierungstest nach OECD-Prüfrichtlinie 406 an 20 P-Strain-Meerschweinchen mit einem Handelsprodukt, welches hauptsächlich aus einem **C9-Aromatengemisch** bestand (darunter 32 % 1,2,4-Trimethylbenzol und 9 % 1,3,5-Trimethylbenzol) lieferte ein negatives Testergebnis. Die intradermale Induktion erfolgte mit einer 0,1%igen (G/V) Testzubereitung in Maiskeimöl, die topische Induktion mit einer 50%igen (G/V) Testzubereitung in Maiskeimöl. Auf die Provokation mit einer 25%igen (G/V) Testzubereitung in Maiskeimöl reagierte nach 24 und 48 Stunden keines der 20 Tiere (ECHA 2023).

Ein weiterer, sekundär zitierter Maximierungstest in Anlehnung an OECD-Prüfrichtlinie 406 an 20 Dunkin-Hartley-Meerschweinchen mit höheren Konzentrationen eines weiteren Handelsprodukts, einem **C9/C10-Aromatengemisch** (84 % (G/G) C9-Aromaten (Trimethylbenzole und Ethyltoluole), 15 % (G/G) C10-Aromaten (Dimethylethylbenzole (im Dossier fälschlich als Diethylethylbenzole bezeichnet) und Diethylbenzole), 1 % (G/G) C8-Aromaten (Xylole) und Benzol), lieferte ein formal positives Ergebnis. Dabei erfolgte die intradermale Induktion mit einer 5%igen (G/V) Testzubereitung in Maiskeimöl, die topische Induktion mit der unverdünnten Testsubstanz und die Provokation abweichend von der Prüfrichtlinie statt mit der höchsten nicht irritierenden Konzentration mit drei Konzentrationen von 5 bis 50 % (G/V) in Maiskeimöl. Die Ergebnisse sind in Tabelle 6 zusammengefasst. Insgesamt wurde eine geringe Sensibilisierungsquote festgestellt, wobei auch Irritationen beobachtet wurden. Bei der sieben Tage nach der ersten erfolgten zweiten Provokation wurde eine moderate Sensibilisierungsquote festgestellt (ECHA 2023). Der Test ist als nicht valide zu bewerten, da die Wahl der Konzentrationen nicht nach Prüfrichtlinie erfolgte und nicht nachvollziehbar ist. Mit der 50%igen Testzubereitung wurden Irritationen beobachtet, sodass dies nicht die höchste nicht irritierende Konzentration ist. Eine Dosis-Wirkungs-Beziehung lässt sich nicht erkennen.

**Tab. 6:** Ergebnis des Maximierungstests mit 1,2,4-Trichlorbenzol (ECHA 2023)

**Konzentrationen (G/V)**	**Anzahl positiver Reaktionen bei**			
	**induzierten Tieren**		**nicht induzierten Tieren**	
	**Ablesung ** **nach 24 h **	**Ablesung ** **nach 48 h **	**Ablesung ** **nach 24 h **	**Ablesung ** **nach 48 h**
erste Provokation				
5 %	11/20	10/20	1/10	0/10
25 %	3/20	2/20	1/10	0/10
50 %	7/20	1/20	3/10	0/10
zweite Provokation				
5 %	8/20	7/20	0/10	0/10
50 %	9/20	6/20	0/10	0/10

##### 5.4.1.2 Untersuchungen mit New Approach Methods (NAMs)

Die Vorhersage des hautsensibilisierenden Potenzials mit einem statistischen quantitativen Struktur-Wirkungs-Beziehungs-Modell (OECD QSAR Toolbox, Version 4.7) war für alle drei Konstitutionsisomere negativ. Weitere Daten aus nicht-tierbasierten Alternativverfahren (NAMs) liegen nicht vor, daher lässt sich kein Gesamtergebnis aus NAMs ableiten (OECD 2024).

#### 5.4.2 Atemwegssensibilisierende Wirkung

Hierzu liegen keine Daten vor.

### 5.5 Reproduktionstoxizität

#### 5.5.1 Fertilität

Im Nachtrag von 1998 (Greim 1998) ist eine 3-Generationen-Studie beschrieben:

Das in der Generationenstudie eingesetzte Gemisch aus **C9-Aromaten** wies folgende Zusammensetzung auf: 40,5 % 1,2,4-Trimethylbenzol; 8,37 % 1,3,5-Trimethylbenzol; 6,18 % 1,2,3-Trimethylbenzol; 2,74 % Cumol; 3,2 % *o*-Xylol; 3,97 % *n*-Propylbenzol; 7,05 % 4-Ethyltoluol; 15,1 % 3-Ethyltoluol; 5,44 % 2-Ethyltoluol; 6,19 % ≥ C10-Aromaten. Der Gesamt-Trimethylbenzolanteil betrug 55 %. COBS-CD-Ratten wurden gegen dieses C9-Aromaten-Gemisch in den Konzentrationen 0; 103 ± 2,1; 495 ± 69 oder 1480 ± 20,5 ml/m^3^ ganzkörperexponiert (0, 57, 273 oder 815 ml Gesamt-Trimethylbenzol/m^3^). Bei den Elterntieren der F0- und F1-Generation zeigte sich ab der mittleren Konzentration eine statistisch signifikante Verzögerung der Körpergewichtsentwicklung, begleitet von einer verminderten Futteraufnahme. Bei den Elterntieren der F2-Generation war ab der niedrigsten Konzentration eine konzentrationsabhängig reduzierte Körpergewichtszunahme (ca. 10 %) zu beobachten. Bei der höchsten Konzentration waren in der F1-Generation die Wurfgröße sowie der männliche Fertilitätsindex vermindert. In den beiden anderen Generationen jedoch waren weder Wurfgrößen noch Fertilitätsindices verändert. Eine NOAEC für Parentaltoxizität ließ sich für die F2-Generation nicht ableiten, für die F0- und F1-Generation lag die NOAEC für Parentaltoxizität bei 103 ml C9-Aromaten-Gemisch/m^3^ (57 ml Gesamt-Trimethylbenzol/m^3^). Die NOAEC für die Fertilitätsbeeinträchtigung betrug 495 ml C9-Aromaten-Gemisch/m^3^ (273 ml Gesamt-Trimethylbenzol/m^3^). Die unterschiedliche Empfindlichkeit bei der parentalen Toxizität zwischen der F2- und der F0- bzw. F1-Generation erklärt sich vermutlich durch das geringere Alter der F2-Generation zu Expositionsbeginn. In einer Nachbewertung wurde ein Substanzeffekt auf die Fertilität in Frage gestellt, da die männliche Fertilität nur in der F1-Generation beeinträchtigt war und nicht in der F0- und F2-Generation (Greim 1998; McKee et al. 1990). Der Vergleich zwischen den drei Generationen wird jedoch dadurch erschwert, dass das Alter der Tiere bei Expositionsbeginn in den drei Generationen unterschiedlich war (Greim 1998).

Neue Daten zu diesem Endpunkt liegen nicht vor.

#### 5.5.2 Entwicklungstoxizität

Der Nachtrag von 1998 (Greim 1998) beinhaltet eine Studie zur pränatalen Entwicklungstoxizität nach OECD-Prüfrichtlinie 414:

Trächtige CD1-Mäuse wurden gegen Konzentrationen von 0; 102 ± 2,5; 500 ± 3,7 oder 1514 ± 22,9 ml/m^3^ des in der 3-Generationen-Studie eingesetzten **C9-Aromaten-Gemisches** täglich sechs Stunden lang vom 6. bis zum 15. Gestationstag ganzkörperexponiert (0, 56, 275 oder 833 ml Gesamt-Trimethylbenzol/m^3^). Bei der höchsten Konzentration kam es bei den Muttertieren zu erhöhter Mortalität, verzögerter Körpergewichtsentwicklung, verminderter Futteraufnahme sowie zu Effekten auf hämatologische Parameter. Die Feten wiesen bei dieser Konzentration eine erhöhte Inzidenz für Gaumenspalten auf, was auf die massive Maternaltoxizität zurückgeführt wurde. Bei der mittleren und der hohen Konzentration war das Fetengewicht pro Wurf statistisch signifikant (7 % bzw. 34 %) reduziert. Darüber hinaus wurden bei der höchsten Konzentration eine statistisch signifikante Erhöhung der Postimplantationsverluste pro Wurf und Ossifikationsverzögerungen festgestellt. Die NOAEC für Entwicklungstoxizität lag bei 102 ml/m^3^ und für Maternaltoxizität bei 500 ml/m^3^ für das C9-Aromaten-Gemisch, entsprechend 56 und 275 ml Gesamt-Trimethylbenzol/m^3^ (Greim 1998; International Research and Development Corporation 1989). Die Studie liegt als Entwurf vor, ein finaler Bericht ist nicht verfügbar. Bei einer Gestationsdauer von 19 bis 20 Tagen kommt es bei Mäusen aufgrund des Gehirnwachstums zu einem schnellen Schädelwachstum. Das Wachstum in lateraler Richtung führt zu einer Vergrößerung des Spaltes, der durch die Gaumenplatten geschlossen werden muss. Falls die Rotation der Platten verspätet oder beeinträchtigt ist (z. B. durch Wachstumsverzögerungen beim Embryo) können sich die Platten nicht treffen und daher nicht fusionieren, was zu einer Gaumenspalte führt. Spezies mit einer längeren Gestationsdauer verfügen über ein proportional längeres Fenster für den Verschluss der Gaumen, sodass Entwicklungsverzögerungen keinen derartigen Effekt auf den Schluss der Gaumen zur Folge haben (DeSesso und Scialli 2018). Daher lässt sich aus der Studie von International Research and Development Corporation (1989) an Mäusen kein teratogenes Potenzial für Trimethylbenzol ableiten.

In einer Dosisfindungs-Inhalationsstudie wurde ein **C9**-**Aromatengemisch** eingesetzt, das folgende Zusammensetzung aufwies: 39,18 % 1,2,4-Trimethylbenzol; 8,09 % 1,3,5-Trimethylbenzol; 5,48 % 1,2,3-Trimethylbenzol; 2,76 % Cumol; 3,17 % *o*-Xylol; 3,95 % *n*-Propylbenzol; 6,13 % 4-Ethyltoluol; 15,85 % 3-Ethyltoluol; 5,78 % 2-Ethyltoluol; ca. 8 % C10-Aromaten. Der Gesamt-Trimethylbenzolanteil betrug 53 %. Je fünf verpaarte CD-Mäuse wurden vom 6. bis zum 15. Gestationstag sechs Stunden pro Tag gegen 0, 100, 250, 500, 1000 oder 1500 ml C9-Aromatengemisch/m^3^ (ca. 0, 53, 132, 264, 530 oder 795 ml Trimethylbenzol/m^3^) exponiert. In der höchsten Konzentrationsgruppe verendeten zwei Tiere und der Futterverbrauch war bei den drei überlebenden Tieren reduziert. Das fetale Körpergewicht war ab 500 ml/m^3^ (ca. 264 ml Trimethylbenzol/m^3^) statistisch signifikant erniedrigt (um 14 %, 20 % bzw. 31 %). Nach Meinung der Autoren liegt es im Bereich der historischen Kontrollen von 0,84 g (0,73–0,94 g) (International Research and Development Corporation 1989).

In der bereits beschriebenen 3-Generationen-Studie mit einem **C9-Aromatengemisch** an COBS-CD-Ratten kam es bei der F1-Generation bei der höchsten Konzentration zu einem erniedrigten Gestationsüberlebensindex; in den beiden anderen Generationen jedoch nicht. Die Exposition hatte bis zur höchsten Konzentration keinen Effekt auf die Überlebensindices der Nachkommen am 4. und 21. Postnataltag. In der höchsten Konzentrationsgruppe war die Körpergewichtsentwicklung bei den Nachkommen der F0-, F1- und F2-Tiere konzentrations- und zeitabhängig ab dem 7. Postnataltag verzögert (siehe Abschnitt 5.5.1; Greim 1998; McKee et al. 1990). Für die Bewertung der Perinataltoxizität werden Effekte bis zum 4. Postnataltag berücksichtigt. Daher lässt sich eine NOAEC für Perinataltoxizität von 1480 ml C9-Aromaten-Gemisch/m^3^ (815 ml Gesamt-Trimethylbenzol/m^3^), der höchsten Konzentration, ableiten.

Seither wurde eine weitere Studie zur pränatalen Entwicklungstoxizität veröffentlicht. In dieser Studie, zum Großteil nach OECD-Prüfrichtlinie 414 (Methodik und Ergebnisdarstellung nach Prüfrichtlinie; k. A. zu Anzahl der Aborte sowie der Muttertiere, die früher geworfen haben), wurden 17–24 trächtige Sprague-Dawley-Ratten sechs Stunden pro Tag gegen **1,3,5-Trimethylbenzol** (0, 100, 300, 600 oder 1200 ml/m^3^; Reinheit 99 %) oder **1,2,4-Trimethylbenzol **(0, 100, 300, 600 oder 900 ml/m^3^; Reinheit 99 %) vom 6. bis zum 20. Gestationstag ganzkörperexponiert. Eine statistisch signifikante Abnahme der maternalen Körpergewichtszunahme und der Nahrungsaufnahme trat ab 300 ml/m^3^ 1,3,5-Tri­methylbenzol und 600 ml/m^3^ 1,2,4-Trimethylbenzol auf. Fetale Toxizität, ausgedrückt als statistisch signifikante Verringerung des fetalen Körpergewichts als Zeichen einer Wachstumsverzögerung trat bei beiden Verbindungen ab jeweils 600 ml/m^3^ auf. Bei 1,3,5-Trimethylbenzol war das fetale Körpergewicht nur bei männlichen Tieren um 5–7 % bei 600 ml/m^3^ zum Kontrollwert verringert, sowie bei 1200 ml/m^3^ um 12 % für weibliche und männliche Feten. 1,2,4-Tri­methylbenzol führte zu Körpergewichtsabnahmen im Vergleich zur Kontrollgruppe um 5 % bei 600 ml/m^3^ und um 11–12 % bei 1200 ml/m^3^. Bei diesen Konzentrationen trat bereits Maternaltoxizität auf. Es gab keine Hinweise auf substanzbedingte embryoletale oder teratogene Effekte nach inhalativer Exposition gegen eines dieser beiden Trimethylbenzol-Isomere. Die NOAEC für die maternale Toxizität betrug 100 ml/m^3^ für 1,3,5-Trimethylbenzol und 300 ml/m^3^ für 1,2,4-Trimethylbenzol, und die NOAEC für die Entwicklungstoxizität lag bei jeweils 300 ml/m^3^ für beide Isomere (Saillenfait et al. 2005).

Studien zur Entwicklungsneurotoxizität liegen nicht vor.

### 5.6 Genotoxizität

#### 5.6.1 In vitro

**1,3,5-Trimethylbenzol** wirkte in Salmonella-Mutagenitätstests an den Stämmen TA98, TA100 und TA2637 jeweils mit und ohne Zusatz eines metabolischen Aktivierungssystems bis 200 µg/Platte nicht mutagen (Greim 1998). Auch ein **C9-Aromatengemisch** (8,37 % 1,3,5-Trimethylbenzol; 40,5 % 1,2,4-Trimethylbenzol; 6,18 % 1,2,3-Trimethylbenzol) war weder mutagen in Salmonella-Mutagenitätstests mit TA98, TA100, TA1535, TA1537 oder TA1538 bis 0,5 µl/Platte noch im HPRT-Genmutationstest mit CHO-Zellen und führte nicht zu erhöhten SCE sowie Chromosomenaberrationen (Greim 1998).

Keine Mutagenität trat im Salmonella-Mutagenitätstest mittels Platteninkorporation mit den Stämmen TA97a, TA98, TA100 oder TA102 bei 0,1–30 µl **1,2,4-Trimethylbenzol**/Platte und 0,1–40 µl **1,3,5-Trimethylbenzol**/Platte auf. Die jeweils höchste Konzentration war zytotoxisch (bestimmt als Reduktion der Revertantenzahl unter 75 % der Kontrollzahlen bzw. im Hintergrund-Bakterienrasen). Auch im Präinkubationstest waren 1,2,4- und 1,3,5-Trimethylbenzol nicht mutagen in TA98 und TA100 jeweils mit und ohne Zusatz eines metabolischen Aktivierungssystems. Nur **1,2,3-Tri­methylbenzol** wirkte als einzige Verbindung im Platteninkorporationstest mutagen. Der stärkste Effekt wurde in TA97a gemessen (Janik-Spiechowicz et al. 1998). Die Reinheit des 1,2,3-Trimethylbenzols ist nicht angegeben. Für das vom selben Hersteller bezogene Produkt wird eine Reinheit von 90–95 % (Korsak et al. 1997; Korsak und Rydzyński 1996) ohne Angabe von Verunreinigungen angeführt. Effekte traten nur ab der von der aktuellen OECD-Prüfrichtlinie 471 vorgegebenen maximalen Testkonzentration von 5 µl/Platte auf. Die Mutagenität von 1,2,3-Trimethylbenzol trat nur ohne metabolische Aktivierung auf, was nicht plausibel erklärt werden kann. Strukturanaloge Substanzen (Xylol, Cumol, Ethylbenzol etc.) sind nicht mutagen. Die Studie wird nicht zur Bewertung herangezogen.

Ein weiteres **Gemisch** (Farbasol, 96 % C9-Alkylbenzole, davon 46 % Trimethylbenzole, 40 % Ethyltoluole) zeigte mit und ohne Zusatz eines metabolischen Aktivierungssystems keine mutagene Wirkung im Salmonella-Mutagenitätstest mit den Stämmen TA97a, TA98, TA100 oder TA102 bei 5–30 µl Farbasol/Platte. Bei 30 µl/Platte trat Zytotoxizität in allen Stämmen außer TA97a auf (Janik-Spiechowicz und Wyszyńska 1998). TA97a wurde nicht bis zu toxischen Konzentrationen getestet.

Salmonella-Mutagenitätstests mit den Stämmen TA98, TA100, TA1535, TA1537 und TA1538 wurden mit einem **C9-Gemisch** aus Trimethylbenzol und Ethyltoluol (k. A. der Trimethylbenzol-Konzentration) durchgeführt. Die Konzentrationen von 2–200 µl/Platte führten mit oder ohne Zugabe eines metabolischen Aktivierungssytems im Platteninkorporationstest nicht zu Mutagenität. Bakteriotoxizität wurde im Vorversuch an TA100 ab 200 µl/Platte als stark reduzierter Bakterienrasen festgestellt (FMC Corp 1984). Zytotoxizität wurde für alle Bakterienstämme maximal in einem von zwei Ansätzen erreicht.

#### 5.6.2 In vivo

Je fünf männlichen BALB/c-Mäusen wurden eine Stunde nach der subkutanen Implantation von Bromdesoxyuridin (BrdU)-Tabletten 0, 20, 40, 60 oder 80 % der LD_50_-Menge des jeweiligen Trimethylbenzol-Isomers intraperitoneal injiziert. Das entspricht 0, 730, 1470, 2200 oder 2970 mg **1,2,3-Trimethylbenzol**/kg KG und 0, 900, 1800, 2700 oder 3600 mg/kg KG der beiden weiteren **Trimethylbenzol-Isomere**. Die jeweils höchste Dosis war aufgrund zu hoher Knochenmarkstoxizität oder frühzeitigem Verenden der Tiere nicht auswertbar. Nach 23 Stunden wurden statistisch signifikant erhöhte SCE-Häufigkeiten in Knochenmarkszellen ab 730 mg 1,2,3-Trimethylbenzol/kg KG, ab 900 mg 1,2,4-Trimethylbenzol/kg KG sowie ab 1800 mg 1,3,5-Trimethylbenzol/kg KG beobachtet. Bei 1,2,3-Trimethylbenzol betrug die Anzahl an SCE/Zelle 4,14 ± 0,17 bei 730 mg/kg KG und 4,73 ± 0,24 bei 2200 mg/kg KG. Die Werte der Tiere, die als Negativkontrolle Mineralöl („light white oil“) erhielten, betrugen 3,70 ± 0,12 SCE/Zelle. Für 1,2,4-Trimethylbenzol und 1,3,5-Tri­methylbenzol betrugen die SCE-Werte/Zelle 5,46 ± 0,43 bzw. 3,90 ± 0,16 für die Dosis von 900 mg/kg KG und 6,61 ± 0,25 bzw. 4,58 ± 0,29 für die Dosis von 2700 mg/kg KG. In der Gruppe der Tiere, die als Negativkontrolle Mineralöl („light white oil“) erhielten, waren die jeweiligen Häufigkeiten 4,29 ± 0,13 und 3,58 ± 0,18 SCE/Zelle. Als Positivkontrolle induzierten 2 mg Mitomycin C/kg KG 21,34 ± 1,36 SCE/Zelle (Janik-Spiechowicz et al. 1998). Für jedes der Isomere betrug die maximale SCE-Häufigkeit also nur das etwa 1,5-Fache des jeweiligen Kontrollwerts. Hier sind die Zahlenwerte aus dem Text wiedergegeben. Die Angaben in Text und Abbildung sind jedoch widersprüchlich.

Eine einmalige orale Gabe von 6,2–780 mg **1,2,4-Trimethylbenzol**/kg KG führte bei Mäusen zu keiner erhöhten Anzahl von Mikronuklei in polychromatischen Erythrozyten aus dem Knochenmark (Greim 1998).

Männliche und weibliche BALB/c-Mäuse (je 4/Dosis und Zeitpunkt; im ersten Versuch wurden nur männliche Tiere mit der jeweils höchsten Dosis behandelt, dort beträgt die Tierzahl entsprechend je 8/Dosis und Zeitpunkt, weil sie zusammen ausgewertet wurden) erhielten eine zweimalige intraperitoneale Gabe (Intervall 24 Stunden) der Trimethylbenzole. Die Gesamtdosis entsprach bei männlichen Tieren 40 % oder 80 % der LD_50_ (1470 bzw. 2940 mg **1,2,3-Trimethylbenzol**/kg KG, 2000 bzw. 4000 mg **1,2,4-Trimethylbenzol**/kg KG, 1800 bzw. 3600 mg **1,3,5-Trimethylbenzol**/kg KG) und bei weiblichen Tieren 80 % der LD_50_ (2160 mg 1,2,3-Trimethylbenzol/kg KG, 3280 mg 1,2,4-Trimethylbenzol/kg KG, 2960 mg 1,3,5-Trimethylbenzol/kg KG). Knochenmarkszellen wurden 30, 48 oder 72 Stunden nach der letzten Gabe entnommen. Die Tiere, die als Negativkontrolle Mineralöl („light white oil“) erhielten, wurden nur nach 48 Stunden untersucht. Von jeder Maus wurden die Mikronuklei in 1000 polychromatischen Erythrozyten (PCE) ausgezählt sowie das Verhältnis von PCE zu normochromatischen Erythrozyten (NCE) bestimmt. Bei den Trimethylbenzolen wurde keine Zunahme an Mikronuklei beobachtet, während Mitomycin C als Positivkontrolle eine deutliche Zunahme verursachte. Das Erreichen des Knochenmarks konnte für die männlichen Tiere, die 4000 mg 1,2,4-Trimethylbenzol/kg KG oder 3600 mg 1,3,5-Tri­methylbenzol/kg KG erhielten, anhand der Abnahme des PCE/NCE-Verhältnisses gezeigt werden. Eine zeitabhängige Abnahme des PCE/NCE-Verhältnisses, die nicht statistisch signifikant war, trat nach Verabreichung von 2940 mg 1,2,3-Tri­methylbenzol/kg KG bei den männlichen Tieren auf. Die Dosis von 1470 mg 1,2,3-Trimethylbenzol/kg KG überlebten 72 Stunden nach Behandlung nur drei Mäuse, die von 4000 mg 1,2,4-Trimethylbenzol/kg KG sieben. Das Erreichen des Knochenmarks konnte bei den weiblichen Tieren nicht nachgewiesen werden (Janik-Spiechowicz et al. 1998). Die ausgewertete Anzahl von vier (drei für 1,2,3-Trimethybenzol bei 72 Stunden) Tieren pro Untersuchungszeitpunkt ist zu gering für eine verlässliche statistische Auswertung.

##### Gemische

**Farbasol** (96 % C9-Alkylbenzole, davon 46 % Trimethylbenzole, 40 % Ethyltoluole) führte nach intraperitonealer Injektion von 0, 750, 1500, 2250, 2640 oder 3000 mg/kg KG an männlichen BALB/c-Mäusen zu statistisch signifikant gering erhöhten SCE ab der niedrigsten Dosis, während reines Ethyltoluol statistisch signifikant erhöhte SCE erst ab 1980 mg/kg KG bewirkte. Die beiden höchsten Dosen führten zum Verenden der Tiere. Die Durchführung erfolgte wie für die einzelnen Trimethylbenzol-Isomere bei Janik-Spiechowicz et al. (1998) beschrieben (Janik-Spiechowicz und Wyszyńska 1998).

**Farbasol** verursachte keine Zunahme an Mikronuklei nach zweimaliger intraperitonealer Gabe (Intervall 24 Stunden) mit insgesamt 1500 oder 3000 mg/kg KG an je vier bis acht männliche BALB/c-Mäuse oder 3250 mg/kg KG an je vier weibliche BALB/c-Mäuse, jeweils pro Dosis und Zeitpunkt. Untersucht wurde 30, 48 und 72 Stunden nach erster Gabe. Zytotoxizität in Form eines reduzierten PCE/NCE-Verhältnisses trat nicht auf (Janik-Spiechowicz und Wyszyńska 1998).

Bei Sprague-Dawley-Ratten, die fünf Tage gegen ein **C9-Aromatengemisch** (40,5 % 1,2,4-Trimethylbenzol; 8,37 % 1,3,5-Trimethylbenzol; 6,18 % 1,2,3-Trimethylbenzol) in Konzentrationen von 153 ± 9,6 ml/m^3^, 471 ± 13,1 ml/m^3^ oder 1540 ± 48 ml/m^3^ exponiert waren, wurde keine Zunahme an Chromosomenaberrationen beobachtet (Greim 1998).

### 5.7 Kanzerogenität

#### 5.7.1 Kurzzeitstudien

**1,2,4-Trimethylbenzol** in Konzentrationen von 10, 30, 100 oder 300 µM transformierte Embryonal-Zellen des Syrischen Hamsters nicht (Greim 1998; Rivedal et al. 1992).

#### 5.7.2 Langzeitstudien

Je 50 männliche und weibliche Sprague-Dawley-Ratten erhielten per Schlundsonde 0 oder 800 mg **1,2,4-Trimethylbenzol**/kg KG und Tag (Reinheit 99 %) in Olivenöl an vier Tagen (Montag, Dienstag, Donnerstag, Freitag) in der Woche, 104 Wochen lang. Die Tiere wurden ihre gesamte Lebenszeit lang beobachtet. Für die weiblichen Tiere wurde eine geringe und für die männlichen Tiere eine mittlere Abnahme der Überlebenszeit angegeben (k. w. A.). Behandelte männliche Tiere zeigten einen Anstieg der Inzidenz maligner Tumoren im Kopfbereich von 2 % auf 10 % und der von gutartigen und malignen Tumoren von 54 % auf 62 %. Auffällig sind zwei weibliche Tiere und ein männliches Tier, die ein Neuroesthesioepitheliom entwickelten. Neuroesthesioepitheliome entstehen aus dem olfaktorischen Neuroepithel und treten bei Sprague-Dawley-Ratten selten spontan auf (k. w. A.; Maltoni et al. 1997).

##### Gemisch

Je 25 männliche und weibliche Wistar-Ratten wurden zwölf Monate gegen 0, 94, 194 oder 366 ml **C9-Aromatengemisch** (44,8 % Trimethylbenzol)/m^3^ an fünf Tagen in der Woche sechs Stunden pro Tag exponiert. Die Expositionskonzentrationen entsprechen 0, 42, 87 oder 164 ml Trimethylbenzol/m^3^. In der höchsten Expositionsgruppe hatte ein weibliches Tier ein Leiomyom an der Gebärmutter und ein männliches Tier ein malignes Lymphom an der Milz. Ein männliches Tier, das niedrig exponiert war, wies zudem ein Glioblastom am Cerebellum auf (Clark et al. 1989).

## 6 Bewertung

Das grundlegende Vorgehen zur Bewertung eines Arbeitsstoffes ist der MAK- und BAT-Werte-Liste zu entnehmen (DFG 2025).

Kritische Effekte sind bei Ratten nach Inhalation Neurotoxizität, Lungeneffekte und Einfluss auf die Körpergewichtsentwicklung sowie bei hohen Konzentrationen Reizwirkungen.

Die drei Isomere von Trimethylbenzol zeigen ähnliche toxische Effekte, die auch in gleich hohen Konzentrations- bzw. Dosisbereichen auftreten. Da von einer ähnlichen Wirksamkeit ausgegangen werden kann, werden die Isomere gemeinsam bewertet.

**MAK-Wert. **Bewertungsrelevante Humandaten liegen für Trimethylbenzol nicht vor. Daher werden zur Ableitung eines Grenzwertes die Befunde aus subchronischen Tierversuchen herangezogen.

Der empfindlichste Endpunkt bei Ratten ist nach inhalativer Exposition die Neurotoxizität. Statistisch signifikante geringe Effekte treten ab 25 ml/m^3^ im Hot-Plate-Test (analgetische Wirkung) und ab 100 ml/m^3^ im Koordinationstest (Rotarod-Test) bei 1,2,3-Trimethylbenzol auf. Statistisch signifikante Wirkungen im Hot-Plate-Test und im Rotarod-Test treten ab 100 ml/m^3^ bzw. bei 250 ml/m^3^ mit 1,2,4-Trimethylbenzol auf (Korsak und Rydzyński 1996). Beide Effekte zeigen eine deutliche Konzentrationsabhängigkeit. Die nach vierwöchiger Exposition gegen 25 ml/m^3^ 1,2,3-Trimethylbenzol und 1,3,5-Trimethylbenzol beginnenden geringen Veränderungen an passivem und aktivem Vermeiden waren nicht konzentrationsabhängig (Wiaderna et al. 2002) und werden als nicht advers angesehen. Es bleibt unklar, ob die Testung im Rotarod-Test die Ergebnisse des Hot-Plate-Tests beeinflusst hat. Zudem wurden die Ergebnisse nicht von einer anderen Arbeitsgruppe repliziert und die Adversität der analgetischen Wirkung bei 25 ml/m^3^ ist unklar. Die Konzentration von 25 ml/m^3^ wird als NOAEC angesehen.

In einer Mehrgenerationenstudie führte eine Konzentration von 103 ml C9-Aromaten-Gemisch/m^3 ^(57 ml Gesamt-Trimethylbenzol/m^3^) an juvenilen Ratten zu einer deutlich verzögerten Körpergewichtsentwicklung (McKee et al. 1990). Die Ursache dieser besonderen Sensitivität junger Ratten und die Relevanz für den Menschen sind unklar (Greim 1998).

Da die Effekte zur Neurotoxizität nach vierwöchiger und nach 13-wöchiger Exposition ungefähr gleich stark waren, ist eine Zeitextrapolation nicht erforderlich.

Damit wird ausgehend von der NOAEC von 25 ml/m^3^ mit Übertragung der Daten aus dem Tierversuch auf den Menschen (1:2) und der Berücksichtigung des erhöhten Atemvolumens (1:2) und unter Berücksichtigung des Preferred Value Approach ein MAK-Wert von 5 ml Trimethylbenzol/m^3^ abgeleitet.

**Spitzenbegrenzung. **Da der MAK-Wert anhand der systemischen Wirkung bei längerfristiger Exposition abgeleitet wird, bleiben die Stoffe der Spitzenbegrenzungs-Kategorie II zugeordnet. Die Konzentration von 25 ml/m^3^ wirkt bei zweistündiger Exposition bei Probanden nicht reizend. Darüber hinaus liegen keine Angaben über Reizwirkungen beim Menschen vor. Bei der unzureichenden Datenlage und der kurzen initialen Halbwertszeit der Trimethylbenzole wird der Basisüberschreitungsfaktor von 2 für Stoffe der Kategorie II (Greim 2001) beibehalten.

**Fruchtschädigende Wirkung. **In einer Studie zur pränatalen Entwicklungstoxizität nach OECD-Prüfrichtlinie 414 an CD1-Mäusen mit Ganzkörperexposition gegen ein C9-Aromaten-Gemisch vom 6. bis zum 15. Gestationstag war ab 500 ml/m^3^ das Fetengewicht reduziert. Bei 1514 ml C9-Aromaten-Gemisch/m^3^ kam es zu einer erhöhten Inzidenz an Gaumenspalten und Ossifikationsverzögerungen. Bei dieser Konzentration trat bereits massive Maternaltoxizität in Form von erhöhter Mortalität, verzögerter Körpergewichtsentwicklung, verminderter Futteraufnahme und Effekten auf hämatologische Parameter auf; es wurden auch vermehrt Postimplantationsverluste festgestellt. Die NOAEC für Entwicklungstoxizität lag bei 102 ml/m^3^ und für Maternaltoxizität bei 500 ml/m^3^ für das C9-Aromaten-Gemisch, entsprechend 56 bzw. 275 ml Gesamt-Trimethylbenzol/m^3^ (Greim 1998; International Research and Development Corporation 1989). In einer größtenteils nach gültigen Prüfrichtlinien durchgeführten Studie zur pränatalen Entwicklungstoxizität an Sprague-Dawley-Ratten mit Ganzkörperexposition vom 6. bis zum 20. Gestationstag wurden 1,3,5-Trimethylbenzol und 1,2,4-Trimethylbenzol untersucht. Mit beiden Isomeren traten bei den Feten ab 600 ml/m^3^ verringerte fetale Körpergewichte als Zeichen von Wachstumsverzögerungen auf. Gleichzeitig war Maternaltoxizität in Form einer reduzierten Körpergewichtszunahme und Futteraufnahme zu beobachten. Die NOAEC für Entwicklungstoxizität betrug jeweils 300 ml/m^3^ für beide Isomere und die NOAEC für Maternaltoxizität 100 ml/m^3^ für 1,3,5-Trimethylbenzol und 300 ml/m^3^ für 1,2,4-Trimethylbenzol (Saillenfait et al. 2005). Aus der 3-Generationen-Studie an COBS-CD-Ratten ergibt sich eine NOAEC für Perinataltoxizität von 1480 ml C9-Aromaten-Gemisch/m^3^ (815 ml Gesamt-Trimethylbenzol/m^3^), der höchsten Konzentration. Bei dieser Konzentration kam es bei den Muttertieren zu einer Verzögerung der Körpergewichtsentwicklung sowie zu einer verminderten Futteraufnahme (siehe Abschnitt 5.5.1; Greim 1998; McKee et al. 1990).

Aus den vorliegenden Studien an Ratten und Mäusen lässt sich kein teratogenes Potenzial für Trimethylbenzol ableiten.

Seit 2016 ist für Substanzen, deren MAK-Wert von einem neurotoxischen Effekt abgeleitet wurde, eine Aussage über entwicklungsneurotoxische Effekte beim Fetus notwendig. Studien zur Entwicklungsneurotoxizität liegen jedoch für Trimethylbenzol nicht vor. Daher erfolgt für Trimethylbenzol eine Zuordnung zur Schwangerschaftsgruppe D.

**Krebserzeugende Wirkung. **Nach 104-wöchiger Schlundsondengabe an vier Tagen in der Woche von 0 oder 800 mg **1,2,4-Trimethylbenzol**/kg KG und Tag wurden bei männlichen und weiblichen Sprague-Dawley-Ratten keine erhöhen Tumorinzidenzen beobachtet. Das Auftreten von olfaktorischen Neuroblastomen (Neuroesthesioepitheliom) bei zwei weiblichen Tieren und einem männlichen Tier wird nicht als substanzspezifisch angesehen. Es liegen keine weiteren Daten vor. Die vorliegenden Studien zu Trimethylbenzol ergeben keinen Verdacht auf eine genotoxische Wirkung.

Trimethylbenzol (alle Isomere) wird nicht in eine Kategorie für Kanzerogene eingestuft.

**Keimzellmutagene Wirkung. **Trimethylbenzol ist in vitro nicht mutagen oder klastogen. Eine gering erhöhte (1,5-fach) Anzahl an SCE/Zelle trat in Knochenmarkszellen von männlichen Mäusen nach einmaliger intraperitonealer Applikation auf. Jedoch führte eine zweimalige intraperitoneale oder einmalige orale Gabe bei Mäusen nicht zu einer erhöhten Anzahl an Mikronuklei im Knochenmark. Insgesamt lässt sich somit für Trimethylbenzol keine genotoxische Wirkung ableiten.

Studien an Keimzellen von Säugetieren liegen nicht vor und auch Daten zur Erreichbarkeit der Keimzellen fehlen.

Da aus den In-vivo-Ergebnissen keine mutagene Wirkung in Keimzellen abgeleitet werden kann und keine strukturelle Ähnlichkeit zu In-vivo-Mutagenen besteht, wird Trimethylbenzol (alle Isomere) nicht in eine Kategorie für Keimzellmutagene eingestuft.

**Hautresorption. **Für den Menschen lässt sich aus einer In-vitro-Studie mit humaner Haut eine maximale dermale Aufnahme von 30 mg bei Exposition gegen eine 50%ige **1,2,4-Trimethylbenzol**-Lösung in Ethanol unter Standardbedingungen (eine Stunde Exposition von beiden Händen und Unterarmen, entsprechend 2000 cm² Hautoberfläche) abschätzen (siehe Abschnitt 3.1.3.3). Dies stellt aus zweierlei Gründen einen Worst Case dar: Zum einen enthielt die Rezeptorflüssigkeit Ethanol, was eine Hautpenetration förderte. Zum anderen ist aus Untersuchungen zur Hautirritation (siehe Abschnitt 5.3.1) ersichtlich, dass die maximal nicht-irritierende Konzentration bei 25 % **1,3,5-Trimethylbenzol** liegt und bei einer 50%igen Lösung irritierende Effekte auf der Haut auftraten. In der In-vitro-Studie mit humaner Haut und der Verwendung einer 50%igen Lösung ist somit eine gesteigerte Hautpenetration infolge einer Hautschädigung möglich.

Die systemische NOAEC für Neurotoxizität nach inhalativer Exposition von Ratten gegen Trimethylbenzol (Korsak und Rydzyński 1996, siehe auch Abschnitt 5.2) beträgt 125 mg/m³ (25 ml/m³). Zur toxikokinetischen Übertragung dieser Konzentration auf den Menschen werden berücksichtigt: das Atemvolumen in acht Stunden (10 m^3^), die angenommene inhalative Resorption von 70 % bei der Ratte (analog zur inhalativen Resorption von 70 % beim Menschen (siehe Abschnitt 3.1.1.1; Kostrzewski et al. 1997)), die Übertragung der Daten des Tierversuchs auf den Menschen (1:2) und das erhöhte Atemvolumen am Arbeitsplatz (1:2). Eine Zeitextrapolation ist nicht nötig, da die Effekte mit der Zeit nicht zunahmen. Damit errechnet sich eine systemisch tolerable Menge von 219 mg (125 mg/m^3^ × 0,7 × 10 m^3^ / 2 / 2).

Damit beträgt die Aufnahme über die Haut 30 mg, was 13,7 % entspricht und damit weniger als 25 % der systemisch tolerablen Menge. Die Trimethylbenzole bleiben weiterhin nicht mit „H“ markiert.

**Sensibilisierende Wirkung. **Es liegen keine belastbaren Befunde zur hautsensibilisierenden Wirkung von Trimethyl­benzol beim Menschen vor. Ein valider Maximierungstest am Meerschweinchen mit einem Handelsprodukt lässt kein sensibilisierendes Potenzial erkennen. Zur Ermittlung eines Gesamturteils aus NAMs liegen nicht ausreichend Daten vor. Es liegen keine Studien vor, aus denen eine atemwegssensibilisierende Wirkung abgeleitet werden kann. Es erfolgt daher weiterhin keine Markierung mit „Sh“ oder „Sa“.
